# Connecting tiger (*Panthera tigris*) populations in Nepal: Identification of corridors among tiger‐bearing protected areas

**DOI:** 10.1002/ece3.10140

**Published:** 2023-05-30

**Authors:** Tek Raj Bhatt, J. Guy Castley, Rebecca Sims‐Castley, Hem Sagar Baral, Alienor L. M. Chauvenet

**Affiliations:** ^1^ Centre for Planetary Health and Food Security Griffith University Southport Queensland Australia; ^2^ School of Environment and Science Griffith University Southport Queensland Australia; ^3^ Rebecca Sims Consulting Tamborine Mountain Queensland Australia; ^4^ Zoological Society of London London UK

**Keywords:** connectivity, habitat linkages, landscape conservation, linkage mapper, pinch points, Siwalik

## Abstract

Habitat fragmentation and isolation threaten the survival of several wide‐ranging species, such as tigers, through increased risk from diseases, disasters, climate change, and genetic depression. Identification of the habitat most likely to achieve connectivity among protected areas is vital for the long‐term persistence of tigers. We aimed to improve the mapping of potential transfrontier protected area corridors for tigers by connecting sites within the Terai Arc Landscape in Nepal and to those in India, highlighting targeted conservation actions needed along these corridors to maintain long‐term connectivity. We used least‐cost corridor modeling and circuit theory to identify potential corridors and bottlenecks in the study area. The landscape's resistance to tigers' movement was gathered from expert opinions to inform corridor modeling. We identified nine potential tiger corridors in the Terai Arc Landscape—Nepal that aligned strongly with the remaining intact habitats of the Siwalik landscape, which could facilitate tiger movement. Banke‐Bardia and Chitwan‐Parsa‐Valimiki complexes and Lagga‐Bhagga and Khata corridors were identified as high‐priority conservation cores and corridors. While our model exhibited congruence with most established corridors in the landscape, it has identified the need to enhance existing corridors to improve landscape connectivity. Several pinch points posing an increased risk to connectivity were identified. Most of these were located near the protected area boundaries and along the Nepal–India border. The Siwalik landscape holds the key to long‐term connectivity in the study area; however, immediate conservation attention is needed, particularly at pinch points, to secure this connectivity for tigers. Validation of identified corridors through empirical research and their conservation is a priority.

## Graphical abstract

Identification of the habitat most likely to achieve connectivity among protected areas is vital for the long‐term persistence of tigers. We used least‐cost corridor modeling and circuit theory to identify potential tiger corridors and bottlenecks in the Terai Arc Landscape in Nepal. The Siwalik hills in the study area holds the key to potential connectivity among protected areas; however, immediate conservation attention is needed, particularly at pinchpoints, to secure this connectivity for tigers.

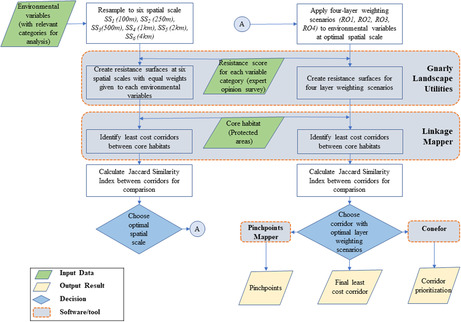

## 1 INTRODUCTION

Protected areas (PAs) are a vital tool for global biodiversity conservation as they support natural habitats relatively undisturbed by human occupation, potentially providing refugia for threatened species (Watson et al., 2014). However, individually, most PAs are considered too small to support viable populations of wide‐ranging species, such as large carnivores (Global Tiger Forum, 2019; Jhala et al., 2021). Moreover, the ongoing transformation of the habitat around PAs for human use has resulted in the further isolation of several PAs, thereby increasing the extinction risk for their inhabitants (Guetté et al., 2018; Ward et al., 2020). The ecological effects resulting from the isolation and small size of PAs can be mitigated by establishing an appropriately connected network of PAs that preserve ecological processes and sustains viable populations of threatened species (Hilty et al., 2020; Wegmann et al., 2014). Despite the call for increased global coverage of well‐connected systems of PAs and effective‐area based conservation measures under Aichi Target 11 of the Convention on Biological Diversity (CBD, 2010), there is currently limited connectivity among PAs in a large proportion of global ecoregions (Saura et al., 2017).

The lack of connectivity among PAs, where most large carnivores are found, is concerning, given their higher sensitivity to ecological effects of habitat fragmentation and isolation (Crooks et al., 2011). Consequently, large carnivores are frequently used as focal species for improving landscape connectivity, given their greater sensitivity to habitat fragmentation, threatened status, ecological importance, charisma, and ability to garner public support for conservation (Hilty et al., 2019; Thornton et al., 2016). One such large carnivore is the tiger, *Panthera tigris*, a globally endangered apex predator and conservation flagship species across large parts of Asia.

Within the past century, tigers lost ~93% of their historical range to humans (Goodrich et al., 2022). Most of the remaining tiger populations (~3900 individuals) are found in small, isolated PAs, increasing the risk of extinction due to genetic inbreeding and depression, natural disasters, disease, poaching, illegal wildlife trade, human persecution, habitat destruction, and climate change threats (Goodrich et al., 2022; GTI, 2011; Jhala et al., 2021). The local extinction of tigers from some PAs has demonstrated that isolated and small PAs are insufficient to conserve the species and highlighted the importance of preserving connectivity among tiger‐bearing PAs (Chundawat et al., 2016). Consequently, there has been growing research on identifying potential connectivity among PAs supporting tiger populations, particularly in the Asian sub‐continent (Dutta et al., 2016, 2018; Rathore et al., 2012; Sharma et al., 2013a, 2013b; Thatte et al., 2018). Identifying and improving connectivity among tiger habitats has been identified as a high‐priority conservation goal for ensuring the long‐term survival of tigers (Jhala et al., 2021; Thatte et al., 2018) and has been included as a key conservation action in the Global Tiger Conservation Action Plan (Global Tiger Forum, 2019; GTI, 2011).

Within Nepal, the tiger population is almost exclusively found within PAs and associated transboundary corridors in the Terai Arc Landscape (TAL) (Chanchani et al., 2014; DNPWC & DFSC, 2022), which are presumed to have reached their carrying capacity (Aryal et al., 2016; Bhandari et al., 2019). For example, between 2010 and 2022, the average tiger density within the country's PAs increased by more than 126% (from 4.6 tigers/100 km^2^ to 10.4 tigers/100 km^2^) while the available protected habitat for tigers only increased by 25% (Dhakal et al., 2014; DNPWC & DFSC, 2022; Karki et al., 2009). There are already signs of increased competition for limited space and food among tigers in Chitwan National Park, where several incidences of tiger mortalities arising from territorial disputes among male tigers have been reported since 2018 (DNPWC, 2020; DNPWC & DFSC, 2018, 2022). Given the rising trend of tiger population numbers in some of these PAs, tigers are likely to move out of the PAs in search of new territory, exacerbating the human–wildlife conflict (Dhungana et al., 2016, 2018) and negative perceptions toward wildlife conservation and PA management (Ramesh et al., 2019).

While transboundary corridors connecting PAs of Nepal and India have been shown to facilitate the tiger movement and decrease the human–wildlife conflict (Chanchani et al., 2014; Wegge et al., 2018), functional corridors connecting tiger‐bearing PAs in Nepal are largely missing. Therefore, improving connectivity among PAs to accommodate more tigers, prevent potential human–tiger conflict, and secure the long‐term persistence of tigers in the landscape has been identified as a major conservation target in the Tiger Conservation Action Plan for Nepal, 2015–2020 (DNPWC, 2016) and the Strategy and Action Plan 2015–2025 for the Terai Arc Landscape, Nepal (MFSC, 2015).

Several connectivity studies, conservation plans, and policies have focused on the identification and protection of corridors among tiger‐bearing PAs to facilitate the safe movement of tigers across the landscape (Dutta et al., 2016; Global Tiger Forum, 2019; Harihar et al., 2018; Jhala et al., 2021; Schoen et al., 2022; Smith et al., 2003; Thinley et al., 2021; Wikramanayake et al., 2004). Specifically for the TAL, previous studies employing cost‐distance and individual‐based movement models have assessed the structural and functional connectivity among protected areas (Kanagaraj et al., 2013; Wikramanayake et al., 2004). These studies identified several corridors and barriers in the landscape for tiger movement. However, the study based on the cost‐distance model was carried out nearly two decades ago, relying on limited computational capabilities and the availability of spatial data layers for environmental variables at the time. In addition, Kanagaraj et al. (2013) did not specifically focus on identifying the locations of the bottlenecks within the corridors and critical features to locate and address to secure structural and functional connectivity.

Given the impact that increased human population and associated development activities may have had on landscape connectivity over the last decade, it is important to reassess the connectivity among PAs of the TAL. We developed a landscape‐scale connectivity model for the transboundary PAs network between Nepal and India capitalizing on the advances in connectivity modeling, computational capabilities, and the availability of better spatial data on environmental variables to provide a more recent and refined assessment of corridor status in the landscape. The study also identifies bottlenecks that may have emerged during the last decade, establishes a baseline of corridor characteristics for future comparisons, and provides concurrent management recommendations to secure landscape connectivity. Opportunities to secure connectivity in one of the most important tiger conservation landscapes are highlighted, and evidence for targeted conservation actions needed along these corridors to maintain long‐term connectivity is provided.

## 2 MATERIALS AND METHODS

### 2.1 Study area

**FIGURE 1:**
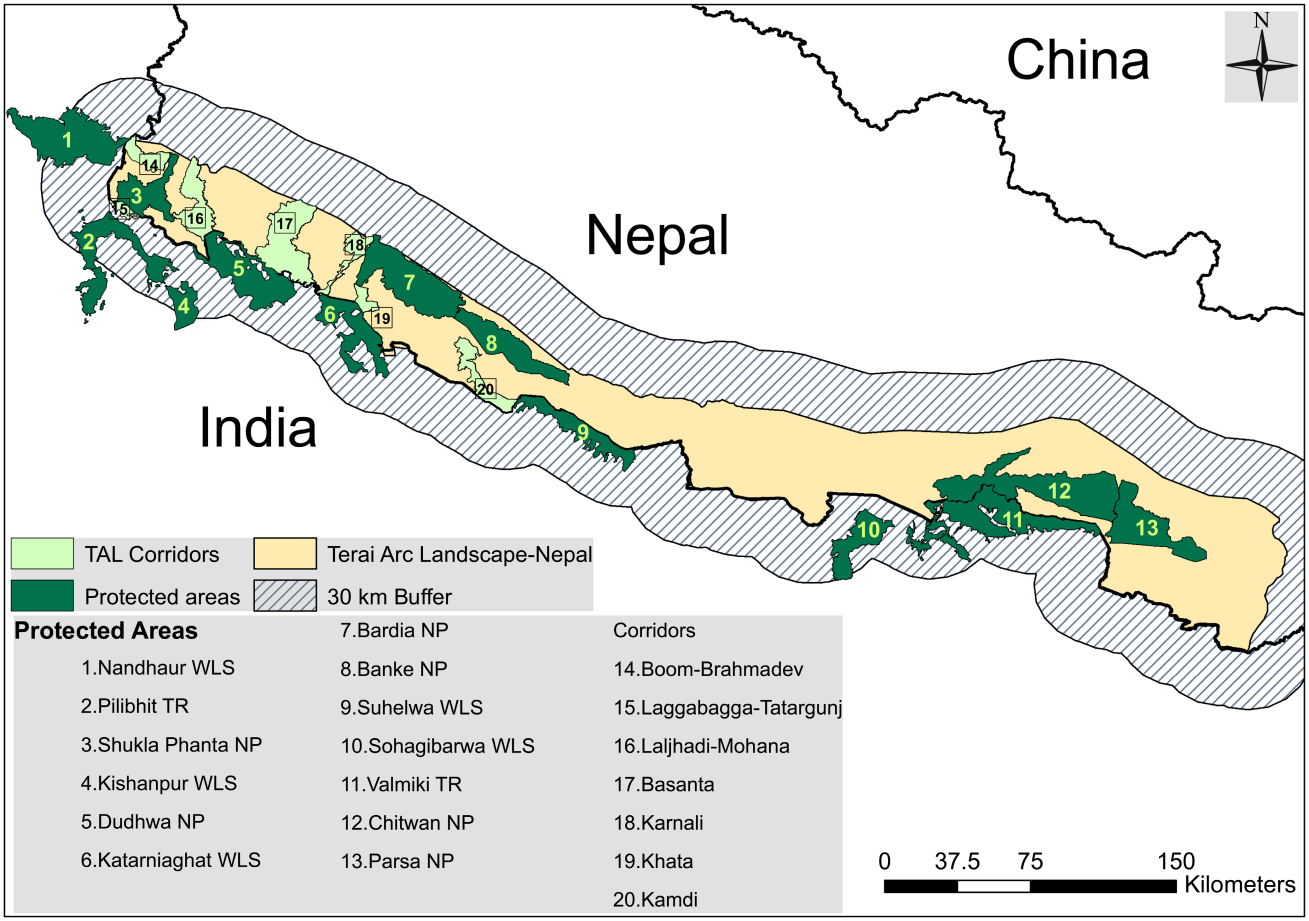
Study area showing the Terai Arc Landscape in Nepal (TAL‐Nepal) along the border with India, extended northern buffer area, tiger‐bearing PAs, and existing transboundary corridors established in the landscape.

The TAL is one of the highest‐priority landscapes for global tiger conservation (DNPWC, 2016; MFSC, 2015). Encompassing an area of ~51,000 km^2^ across India and Nepal, the TAL is home to more than 600 tigers and several other threatened species, such as the Asian elephant, *Elephas maximus*, the greater one‐horned rhino, *Rhinoceros unicornis*, the gaur, *Bos gaurus*, and the gharial, *Gavialis gangeticus* (DNPWC & DFSC, 2022; Jhala et al., 2020; MFSC, 2015). Our study focuses on the TAL in Nepal (TAL‐Nepal) and adjacent PAs in India (TAL‐India) along the Nepal–India border. The TAL‐Nepal covers ~24,710 km^2^ of lowland plains and Himalayan foothills, commonly referred to as the Churia or Siwalik hills in the southwestern part of the country (MFSC, 2015). The spatial extent of our study area encompasses the TAL and an additional 30 km buffer around the TAL to capture the potential transboundary movement of tigers among PAs of India and Nepal as well as beyond the recognized boundaries of the TAL‐Nepal into the higher elevations of the mid‐hills (Figure 1). Selection of a 30 km buffer was based on the reported maximum dispersal distance of radio‐collared adult male tigers in Chitwan National Park (Smith, 1993). The elevation of the study area ranges from 11 to 2931 m above mean sea level.

The study area is characterized by a sub‐tropical to temperate climate and dominant vegetation types, including Sal (*Shorea robusta*) and mixed hardwood forest at low altitudes and pine forest (*Pinus* spp.) at higher altitudes (MFSC, 2015). The low‐lying plains, especially those along the rivers, are characterized by subtropical riverine grasslands and forests and are subject to annual inundation in the wet season. It is one of the country's most productive and densely populated regions, with communities relying heavily on subsistence agriculture for their livelihood (CBS, 2014a).

The study area contains six national parks (NPs), five wildlife sanctuaries (WLSs), and two tiger reserves (TRs) across India and Nepal, all of which are surrounded by a human‐dominated matrix. Although Nepal has five designated tiger‐bearing PAs, they form three distinct tiger subpopulations, with 169 tigers estimated in Chitwan and Parsa NPs, 150 in Banke and Bardia NPs, and 36 tigers estimated in Shukla Phanta NP (DNPWC & DFSC, 2022; Thapa et al., 2018). Chitwan NP shares park boundaries with Valmiki TR in India, forming a single connected tiger sub‐population. Tigers in the Banke NP are connected to Suhelwa WLS via the Kamdi corridor, and the Bardia NP is connected to Katerniaghat WLS via the Khata corridor (Figure 1). The study area also includes seven important transboundary corridors that connect tiger‐bearing PAs in Nepal to adjacent PAs across the border in India (Table 1; Figure 1). The tiger population of the Chitwan NP is connected to Sohagibaruwa WLS via connectivity through Valmik TR. Similarly, Shukla Phanta NP is connected with Nandhaur WLS through the Boom‐Brahmadev corridor, Dudhwa NP through the Laljhadi‐Mohana corridor, and core areas of Pilibhit TR and Kishanpur WLS via the Laggabagga‐Tatargunj corridor forest which is part of the Pilibhit TR. (Figure 1).

**TABLE 1:** PAs and existing corridors in the study area, including information on their geographic location, area of coverage, and estimated tiger population.

Protected area	Country/location	Extent (km^2^)	Estimated tiger population
Parsa NP^a^	Nepal	627	41^a^
Chitwan NP^a^	Nepal	953	128^a^
Banke NP^a^	Nepal	550	25^a^
Bardia NP^a^	Nepal	968	125^a^
Shukla Phanta NP^a^	Nepal	305	36^a^
Valmiki TR^b^	India	901	32^b^
Sohagibarwa WLS^b^	India	428	5^b^ ^,^ ^c^
Dudhwa NP^b^	India	680	20^b^ ^,^ ^c^
Nandhaur WLS^b^	India	380	23^b^ ^,^ ^d^
Katerniaghat WLS^b^	India	401	29^b^
Suhelwa WLS^b^	India	452	0^b^
Pilibhit TR	India	1074	65^b^
Kishanpur WLS	India	227	33^b^ ^,^ ^c^
***Transboundary corridors used by tigers***			
Boom‐Brahmadev Corridor	Shukla Phanta NP—Nandhaur WLS	148	–
Laljhadi‐Mohana Corridor	Shukla Phanta NP—Dudhwa NP	355	1^a^
Laggabagga‐Tatargunj forest block	Shukla Phanta NP—Pilibhit TR	–	–
Karnali River Corridor	Bardia NP—Katerniaghat WLS	150	10^a^
Khata Corridor	Bardia NP—Katerniaghat WLS	202	9^a^
Basanta Corridor	Dudhwa NP—Katerniaghat WLS	652	–
Kamdi Corridor	Banke NP—Suhelwa WLS	450	7^a^

*Note*: The estimated tiger population of sites is based on the national tiger surveys report for Nepal (DNPWC & DFSC, 2022) and India (Jhala et al., 2020).

^a^ National Tiger Survey, Nepal, 2021/2022 (DNPWC & DFSC, 2022).

^b^ National Tiger Survey, India, 2018 (Jhala et al., 2020).

^c^ Number of adult tiger individuals identified from camera trap photographs.

^d^ Nandhaur Wildlife Sanctuary forms part of the larger connected forest patch of Haldwani Forest Division of nearly 1200 km^2^.

### 2.2 Overview of the approach

To identify potential tiger corridors connecting protected areas within the study area, a cost‐distance model was used based on major habitat and anthropogenic variables known to affect the distribution and dispersal of tigers, such as landcover, topography, presence of road networks, and human population density (Dutta et al., 2016; Rathore et al., 2012; Wikramanayake et al., 2004). This approach assumes that environmental variables facilitating species movement have a low cost or resistance, whereas variables that impede movement have a high cost or resistance (Wade et al., 2015; Zeller et al., 2012). It also assumes that the resistance imposed by environmental variables is species‐specific and scale‐dependent (Hilty et al., 2019; Zeller et al., 2012). Although the population density of prey species also influences tiger distribution and movement (Karanth et al., 2004), such information was not readily available at the local landscape scale and, therefore, could not be incorporated into the current corridor modeling.

One of the primary steps in corridor modeling is to develop a travel‐cost resistance surface based on the key environmental variables which influence travel effort, thereby quantifying the expected difficulty the target species encountered in traversing the landscape (Zeller et al., 2012). There are various approaches available to optimize resistance surfaces, including but not limited to expert opinion (Dutta et al., 2016; Rathore et al., 2012), habitat suitability (Balbuena‐Serrano et al., 2022; González‐Saucedo et al., 2021), movement patterns (Carvalho et al., 2016; Proctor et al., 2015), genetic data (Dutta et al., 2022; Jennings et al., 2020), or combinations of these methods (Zeller et al., 2018; Ziółkowska et al., 2016). A resistance surface is generally developed by using empirical data on species distribution or movement in combination with key environmental and anthropogenic variables that may influence the movement of the species (Hilty et al., 2019; Wade et al., 2015). However, when empirical data are unavailable, as it is for many species, researchers have had to rely on expert opinion to compensate for the absence of data on species movement or occurrence (Dutta et al., 2016; Zeller et al., 2012). In the absence of tiger movement and genetic data for our landscape, we used an expert‐based approach to identify the resistance surface for tiger movement in the landscape and the least‐cost corridors. Despite potential limitations in using the expert opinion approach (Poor et al., 2012; Wade et al., 2015), it is still widely regarded as providing a useful estimate of a species' movement potential, especially when coupled with other empirical data (Keeley et al., 2018; Liu et al., 2018).

### 2.3 Creating a resistance surface

#### 2.3.1 Spatial data sources and processing

Vector shapefiles of PAs in the study area were downloaded from the World Database on PAs (https://www.protectedplanet.net) and the National Trust for Nature Conservation geoportal (http://geoportal.ntnc.org.np). Boundaries of Suhelwa WLS, Valmiki, and Pilibhit TRs were digitized from a georeferenced boundaries image file from the ENVIS Centre on Wildlife & Protected Areas database hosted by the Wildlife Institute of India (http://www.wiienvis.nic.in). PAs that shared common boundaries were considered a single core area for corridor mapping.

A landcover layer was created by performing multi‐spectral classification using publicly available Landsat 8 Operational Land Imager (OLI) satellite imagery at 30 m resolution to produce thematic maps identifying the following broad a priori landcover classes: forest, water bodies, agriculture, barren areas, and urban areas. Details on the landcover classification and accuracy assessment are provided in the supplementary file (S1). ASTER‐derived Digital Elevation Model (DEM) data for the study area with a spatial resolution of 30 m was downloaded via the USGS portal (https://earthexplorer.usgs.gov). This dataset was used to generate a slope layer for the study area using the slope tool in ArcGIS.

A road network layer for our study area was generated using data downloaded from OpenStreetMap (OSM) in ESRI‐compatible shapefile format (http://download.geofabrik.de). The data were processed to include only major road classes, such as highways (roads classified as trunks and with names including highways) and other roads (OSM category of primary, secondary, or tertiary roads), which were then converted into a raster grid for corridor analysis.

Human population density was calculated at the lowest level of the administrative division, that is, the Village Development Committee (VDC) for Nepal and Tehsils in India. The projected national population for 2021 based on Nepalese census data 2011 was used to extrapolate the population for each VDC using the ratio method (CBS, 2014b). The ratio method assumes that the sub‐national or local population proportion to the national or larger parent population will remain the same over time and can be calculated using the following equation:

$$p_{t+n}=\frac{p_{t}}{P_{t}}\times P_{t+n}$$

where, *p*_*t*_ and *P*_*t*_ represent sub‐national and national populations during the census year, respectively, and *p*_*t*+*n*_ and *P*_*t*+*n*_ represent projected sub‐national and national populations in the future, respectively.

In the case of India, we used the same method to get the projected population data for 2021 at the sub‐district level from the projected population of the respective state for 2021 based on the Indian national census data 2011 (https://censusindia.gov.in).

#### 2.3.2 Assigning resistance values to variables

Each environmental variable was reclassified into broad categories, which were then assigned resistance values specific to the tiger movement. Categories were guided by previous studies that employed a similar method for tiger connectivity research in India and Nepal (Dutta et al., 2016; Rathore et al., 2012; Wikramanayake et al., 2004). The population density was classified as low (<500 person/km^2^), medium (500–1500 person/km^2^), high (>1500 person/km^2^), or absent. Roads were classified as highways (major roads), other roads (minor roads), or absence. The slope was classified into six categories, from a flat surface (0–2°) to a very steep slope (>35°) (Table 2; Saadat et al., 2008; Zhang & Song, 2020). We conducted expert opinion surveys with five Nepalese tiger experts affiliated with non‐government and academic institutions and with at least ten years of experience in tiger conservation or research to obtain the resistance values (Human research ethics approval from Griffith University ref no. 550/2020) for each category on a scale of 0 to 100, where 0 indicates no resistance to movement, and 100 indicates a complete barrier to movement. We then calculated the average resistance value for each category across the five experts' answers and used these for creating resistance surfaces. (Table 2) using the Resistance and Habitat Calculator in Gnarly Landscape Utilities v0.1.0 in ArcGIS (McRae, Shirk, & Platt, 2013).

**TABLE 2:** Average resistance values derived from expert opinion surveys for each environmental variable.

Type	Environmental variable	Category	Average resistance value
Habitat	Landcover	Water	0
		Forest	17
		Agriculture	67
		Barren	73
		Settlement	100
	Slope	0–2^∘^	0
		2–6^∘^	0
		6–15^∘^	0
		15–25^∘^	0
		25–35^∘^	63
		>35	93
Anthropogenic	Road	Absent	0
		Other roads	65
		Highway	90
	Population density	Absent	0
		Low	64
		Moderate	97
		High	100

### 2.4 Corridor assessment

**FIGURE 2:**
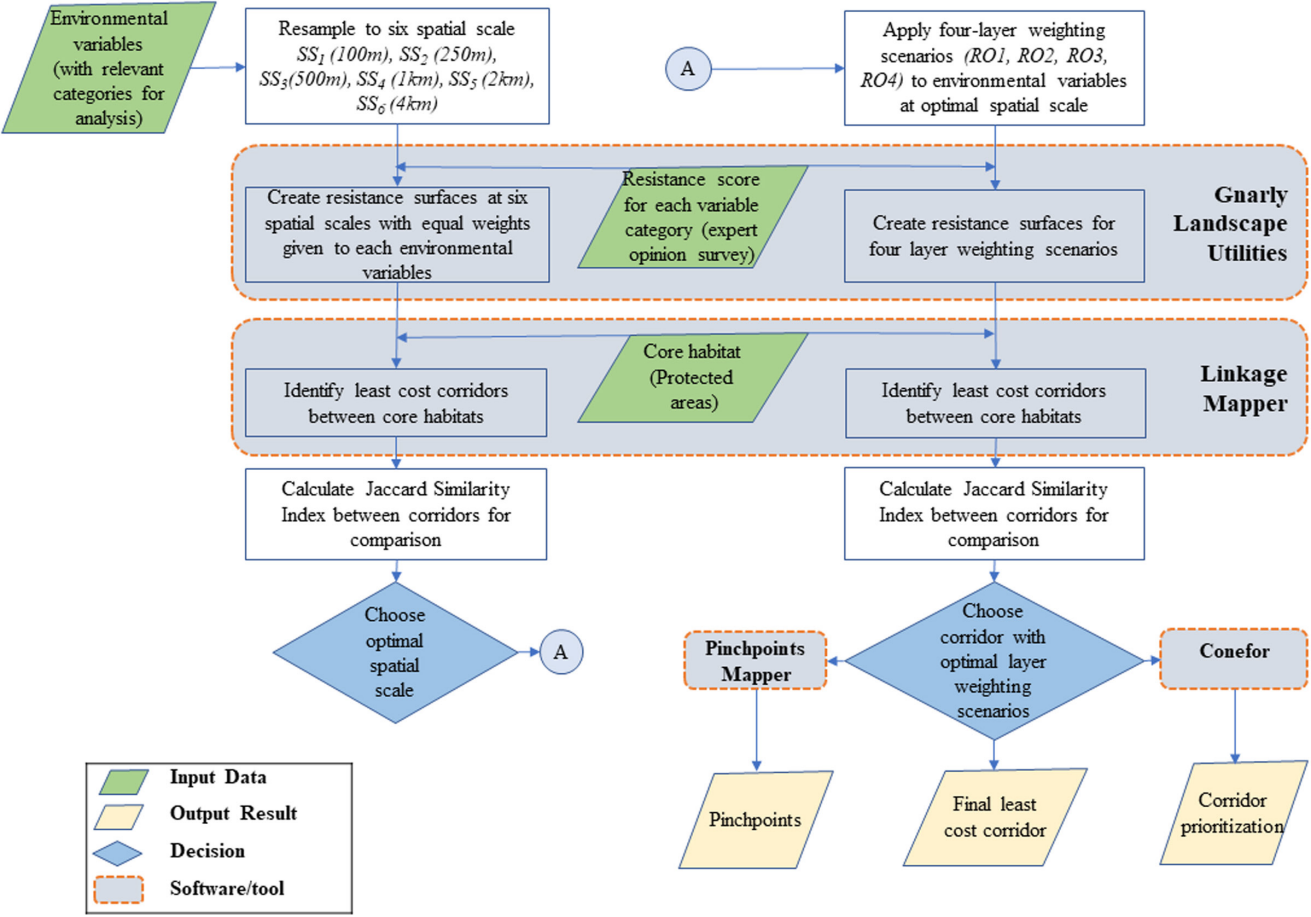
Flowchart of the Corridor Modeling Approach to identify optimal connectivity solutions within the Terai Arc Landscape of Nepal (TAL‐Nepal).

We compared resistance surfaces derived from different spatial scales and weighting scenarios of the input data layers to assess the sensitivity and identify the most optimal spatial scale and layer‐weighting scenario, which were used for mapping the corridor in the landscape (Figures 2 and S2). The Linkage Mapper software (McRae & Kavanagh, 2011) was used to map corridors among the target PAs of the study landscape. Three metrics were calculated to assess the quality and robustness of linkages between PAs; (a) the ratio of cost‐weighted distance to Euclidean distance (CWD:EucD), (b) the ratio of cost‐weighted distance to the least‐cost path (CWD:LCP) and (c) cost‐weighted distance to effective resistance (CWD:Effres).

The CWD:EucD ratio measures the relative length of the corridor compared with the shortest possible distance between target core habitats (in our case, PAs). An ideal corridor will have a CWD:EucD ratio of 1, whereas a higher value indicates the greater difficulty or cost of movement between PAs. The CWD:LCP ratio assesses the corridor quality by measuring the average resistance encountered by tigers per unit distance along a single optimal path between PAs. The Least Cost Path (LCP) represents the path with the lowest cumulative cost of movement between two habitat fragments. Effective Resistance (Effres), calculated in the Linkage Mapper using circuit theory (McRae & Kavanagh, 2011; McRae, Shah, & Mohapatra, 2013), measures the relative isolation of habitat fragments based on the availability of the multiple connecting paths between habitat fragments within a defined corridor area. Therefore while CWD:LCP measures quality along one path only, the CWD:Effres considers corridor robustness more holistically by measuring total resistance along multiple routes between PA pairs, thus making it possible to acknowledge the advantage of multiple or wider routes being present (Dutta et al., 2018; Jones et al., 2015; McRae et al., 2008). When used in combination, these metrics provide a means to assess the overall quality of linkages. For example, a lower value of CWD:LCP indicates a higher quality of the linkages (i.e., lower average resistance per unit distance of the LCP), whereas a higher value of the CWD:Effres indicates a more robust corridor (i.e., more alternative routes present within a corridor area). This implies that linkages with both a low CWD:LCP and a high CWD:Effres are more likely to have a higher potential of aiding tiger movement between PA's.

The Pinchpoint Mapper tool (McRae, 2012), available in the Linkage Mapper plugin in ArcGIS was used to identify the pinch points along the least‐cost corridors. Pinch points are bottlenecks along the corridor with a higher risk of disruption of connectivity or fragmentation of corridors. The Pinchpoint Mapper tool is based on circuit theory and measures the flow of current along the least‐cost corridor to identify bottleneck areas or areas of high resistance. Using Dutta et al. (2016) as a guide, we compared different cutoff widths for a pairwise pinch point analysis along each corridor that connects adjacent PA pairs to determine the most suitable cutoff width for our study.

### 2.5 Conservation prioritization of core habitats and corridors

We calculated the probability of connectivity (*dPC*) using Conefor v2.6 (Saura & Torné, 2009) to assess the importance of each PA (nodes) and the corridors (links) for maintaining the landscape connectivity and assisting with their conservation prioritization. The *dPC* is a graph‐based metric that accounts for both patch size (available habitat) and the probability of dispersal between habitat patches and is frequently used for prioritizing habitat cores and corridors (Ayram et al., 2016; Mohammadi et al., 2021). It is considered relatively more informative than other indices, such as area‐weighted flux or closeness centrality (Baranyi et al., 2011). The index represents the probability that two randomly placed points will fall within interconnected habitat areas of the landscape containing n sets of habitat patches and links connecting those patches. The *dPC* comprises three fractions: *dPC*_intra_, *dPC*_flux_, and *dPC*_connector_, measuring different ways a patch can contribute to connectivity (Kaboodvandpour et al., 2021; Mohammadi et al., 2021). The *dPC*_intra_ is a measure of intrapatch connectivity, and *dPC*_flux_, which depends on the attribute and position of the habitat patch in the landscape, measures how well a particular habitat patch is connected with other patches. The *dPC*_Connector_ depends on the topological position of the habitat patch or links in the landscape and measures its contribution as a stepping stone or link to the dispersal (Saura & Rubio, 2010).

Conefor takes two files as input; a node file representing habitat patches with associated attributes and connection files representing the link between each pair of habitat patches (Saura & Torné, 2009). We used the size and estimated tiger population of each PA separately as habitat node attributes. For preparing the connection input file, we used the CWD among each pair of PAs obtained from the least‐cost corridor analysis to represent the distance between respective pairs of PAs. We compared the influence of various dispersal thresholds (e.g., 30 km, 50 km, 100 km, 150 km, and 200 km) on the overall prioritization of the cores and corridors (S4). However, for the final analysis, we used the maximum EuCD recorded between PA pairs in the least‐cost corridor analysis as the dispersal threshold, that is, between the Chitwan‐Parsa‐Valmiki complex and Banke‐Bardia complex (~175 km), assuming a dispersal probability of 50% for calculating the *dPC* to allow the inclusion of all identified corridors in the prioritization. The average CWD per unit EuCD (~29) among the PA pairs was used to get distance thresholds in CWD (i.e., 5075 km). We used the linkage removal option available in the Conefor, which iteratively removes one linkage at a time to measure its impact on the total connectivity across the landscape to ascertain its importance (Saura & Torné, 2009). The *dPC* metric was used to assess the contribution of habitat cores to habitat availability and maintaining connectivity, whereas *dPC*_connecter_ values were used to prioritize links (i.e., corridors) for their contribution to maintaining connectivity.

## 3 RESULTS

The total area of the landscape included in this study was 66,653 km^2^. The study area included 13 PAs supporting tiger populations, but few were already connected by sharing a common boundary. Therefore, we identified ten disjoint core areas to map corridors in the landscape. The average Euclidean distance between PAs was 51.91 km, with the longest distance between the Chitwan‐Parsa‐Valmiki complex and the Banke‐Bardia complex (173.07 km) and the shortest distance between Shukla Phanta NP and Pilibhit TR (6.37 km).

### 3.1 Selection of spatial scale and layer‐weighting scenario for corridor analysis

**FIGURE 3:**
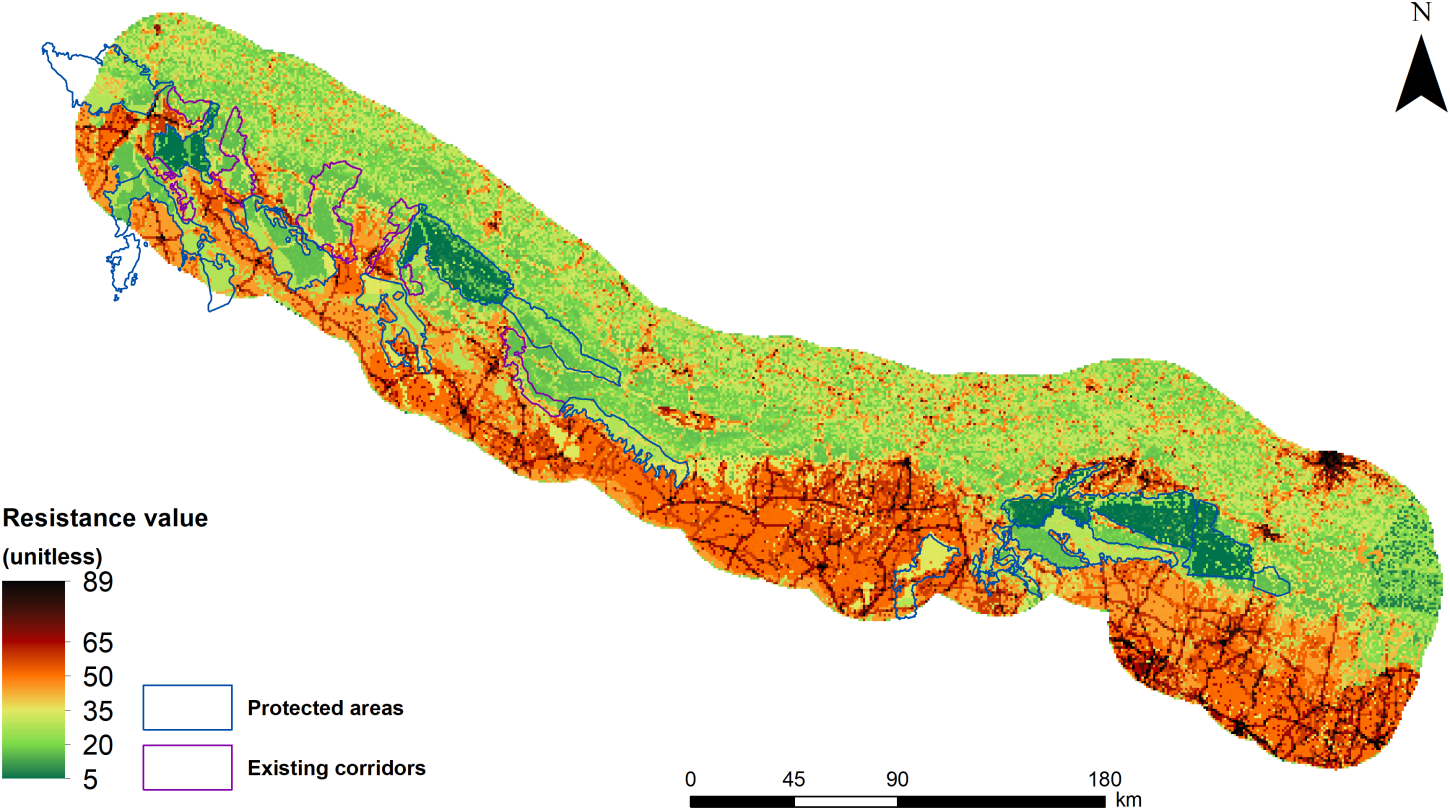
Resistance surface used for the corridor analysis derived using a layer‐weighting scenario where the landcover and human population density layer had twice as much weight as roads and slope layers at 1 km spatial resolution. Resistance values range from 1 (minimum resistance) to 100 (maximum resistance).

Corridor models based on varying spatial scales of input data and layer‐weighting scenarios produced slightly different cost‐weighted distances and least‐cost paths. However, the least‐cost corridors between target PAs had spatial overlap across all models. We identified corridors using a 1 km spatial resolution of input data layers and layer‐weighting scenarios where the landcover and human population density layer had twice as much weight as roads and slope layers to be optimal for our analysis (Figures 3 and S2).

### 3.2 Corridors

Our final corridor dataset contained nine corridors connecting tiger‐bearing PAs within Nepal and adjacent PAs in India (Table 3). Most of the corridors connecting tiger‐bearing PAs of Nepal were identified through the Siwalik landscape. A total of three transboundary corridors connecting the Banke‐Bardia complex to Katerniaghat WLS and Shukla Phanta to Dudhwa and Pilibhit, were identified entirely within the lowland plains. The longest corridor was identified between the Chitwan‐Parsa‐Valmiki complex and the Banke‐Bardia complex (195.85 km), and the shortest corridor was identified between the Banke‐Bardia complex and Katerniaghat WLS (7.41 km).

**TABLE 3:** Different types of distance metrics (in km) measured between each pair of tiger‐bearing PAs of the Terai Arc Landscape Nepal (TAL‐Nepal) and across the border in Terai Arc Landscape India (TAL‐India), together with metrics value (unitless) used to assess the quality of the mapped corridors.

Core 1	Core 2	EucD (km)	CWD (km)	LCP (km)	CWD: EucD	CWD:LCP	CWD:Effres
Chitwan‐Parsa‐Valmiki complex	Banke‐Bardia complex	173.07	3690.35	195.85	21.32	18.84	27.71
Chitwan‐Parsa‐Valmiki complex	Suhelwa WLS	134.34	3143.31	166.64	23.40	18.86	22.86
Banke‐Bardia complex	Suhelwa WLS	10.05	214.90	12.07	**21.39**	17.80	**42.98**
Banke‐Bardia complex	Katerniaghat WLS	7.04	152.23	8.83	21.62	17.24	10.48
Banke‐Bardia complex	Shukla Phanta NP	87.69	1783.14	104.98	20.32	**16.99**	23.14
Banke‐Bardia complex	Dudhwa NP	26.63	1041.29	58.77	39.10	17.72	29.86
Shukla Phanta NP	Dudhwa NP	12.82	487.30	25.56	37.83	19.07	17.67
Shukla Phanta NP	Nandhaur WLS	8.96	406.75	19.90	45.38	20.44	38.90
Shukla Phanta NP	Pilibhit TR	6.37	177.22	8.07	27.80	21.96	27.08

*Note*: The best value for each metric is highlighted in bold letters.

Comparatively, the difficulty of movement for tigers moving through corridors connecting Shukla Phanta NP to other PAs was greater than in corridors connecting the Chitwan‐Parsa‐Valmiki complex and the Banke‐Bardia complex to other PAs. For example, despite having a nearly similar Euclidean distance between Shukla Phanta and Dudhwa NPs and the Banke‐Bardia complex and Suhelwa WLS (12 and 10 km, respectively), the cost of movement through the corridor connecting Shukla Phanta and Dudhwa NPs was much higher than the latter (Table 3). Similarly, despite the long distance between the Banke‐Bardia complex and the Chitwan‐Parsa‐Valmiki complex and between the Banke‐Bardia complex and Shukla Phanta NP, the cost of movement per unit distance (EucD = 173.07, CWD: EucD = 21.32 and EucD = 87.69, CWD: EucD = 20.32) was lower than other pairs of PAs. The resistance along the most suitable path (i.e., least‐cost path) was also found to be lowest for the corridor connecting the Banke‐Bardia complex and Shukla Phanta NP (CWD:LCP = 16.99), Katerniaghat WLS (CWD:LCP = 17.24), Dudhwa NP (CWD:LCP = 17.72) and Suhelwa WLS (CWD:LCP = 17.80). This indicates that it is easier for tigers in the Banke‐Bardia complex to move to other PAs, whereas it is more difficult for tigers in the Shukla Phanta NP.

The CWD:Effres, which measures the robustness of the corridor to being severed, was highest for the corridor connecting the Banke‐Bardia complex to Suhelwa WLS, indicating the presence of multiple low‐cost alternative routes, a wider corridor habitat and more robust connectivity potential between these PAs. Whereas the ratio was lowest for the corridor connecting Banke‐Bardia complex to Katerniaghat WLS, indicating fewer alternative routes available to tigers, a narrow corridor habitat and a higher risk of severing the connectivity. Across all the identified corridors, the ratio was higher for the corridors passing through the Siwalik landscape, indicating that these corridors consist of more low‐resistance alternative paths for tigers to move between PAs and, consequently, are less likely to be severed.

**FIGURE 4:**
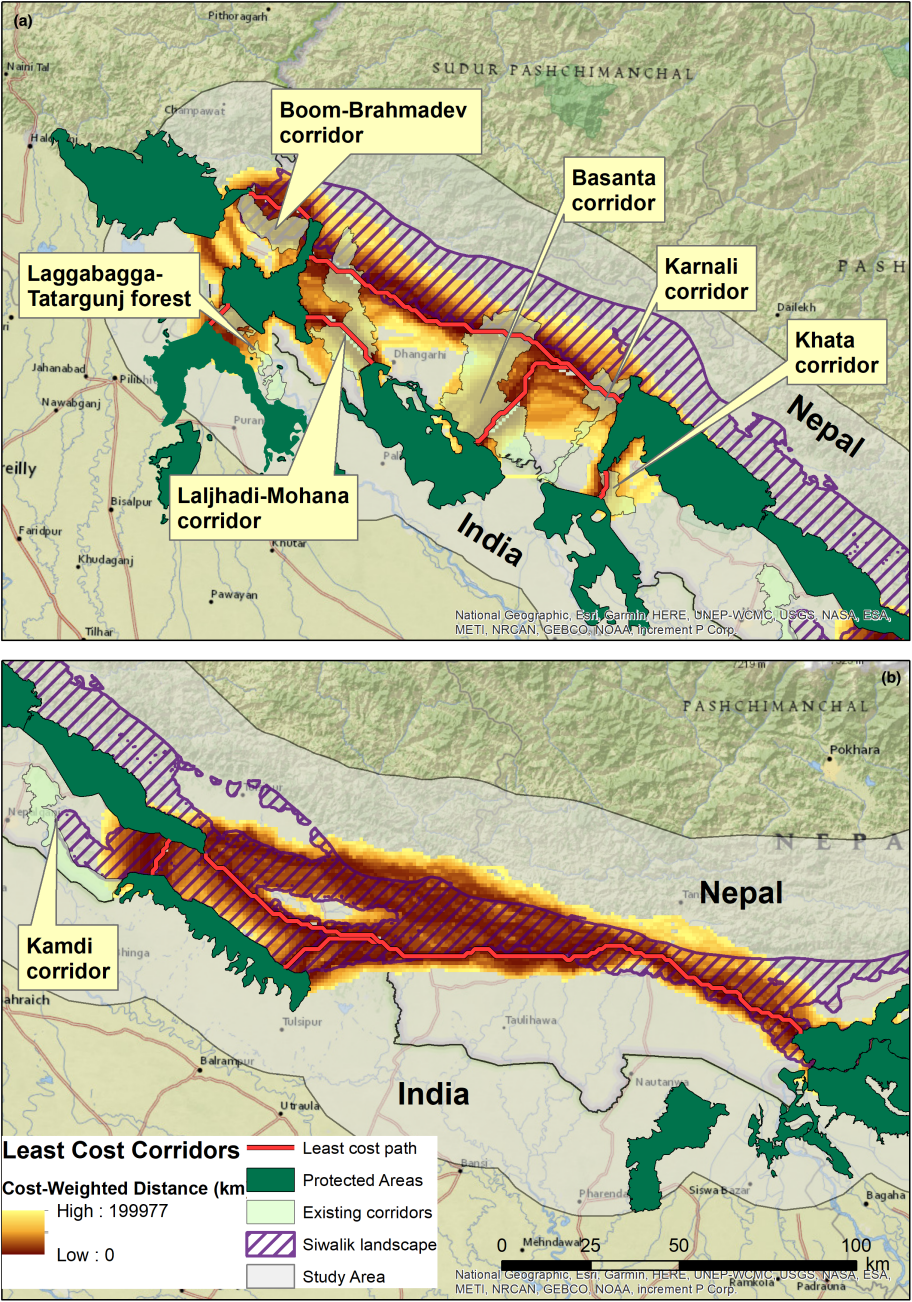
Overlap between identified corridors, existing corridors, and the Siwalik landscape for (a) the western block and (b) the central/eastern block of the study area. The width of the least‐cost corridors is limited to a 200 km cost‐weighted distance.

The corridors modeled for this current study overlapped with the already established corridors in the landscape to varying degrees. While optimal corridors intersected with established corridors in several locations, a significant area outside the boundaries of existing corridors was identified as potential corridor habitat. Most of the optimal corridor habitat between Nandhaur WLS and Shukla Phanta NP was identified north of the existing Boom‐Brahmadev corridor. Similarly, most of the optimal corridor habitat between Katerniaghat WLS and Bardia NP was identified west of Geruwa River outside the Khata corridor. The corridor models also partially identified the Laljhadi‐Mohana and Basanta corridors, allowing opportunities to optimize existing corridors to improve connectivity in the landscape. However, the corridor model completely missed the Kamdi corridor established to connect Banke NP in Nepal to Suhelwa WLS in India. However, an alternative, shorter, least‐cost path was identified between these two transboundary PAs (Figure 4).

### 3.3 Pinch points for corridors

**FIGURE 5:**
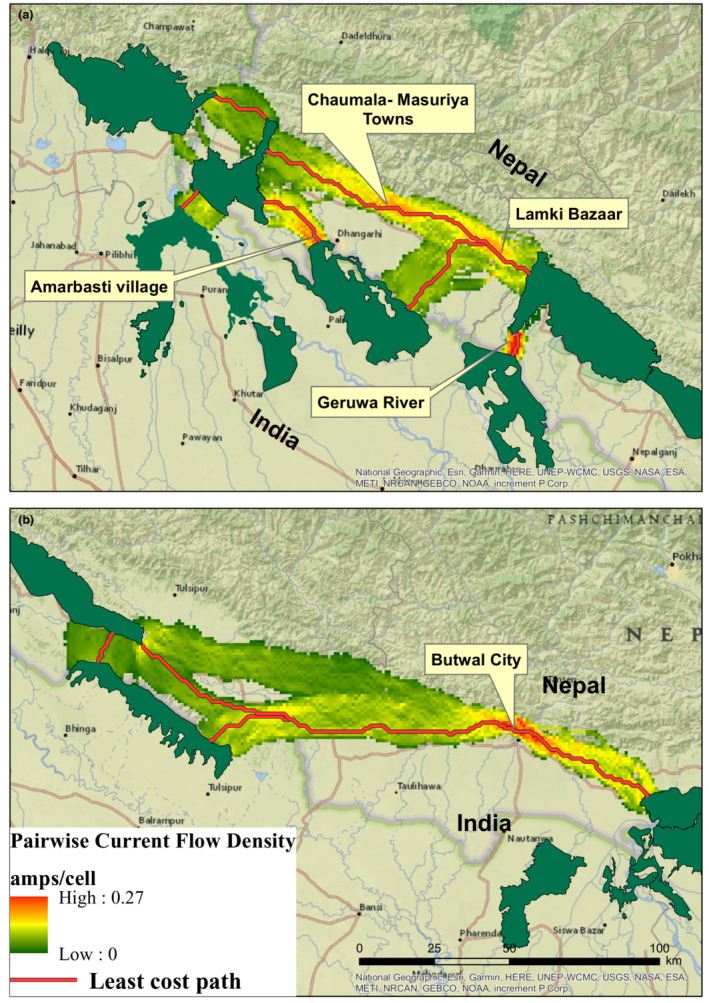
Pinch point areas, indicated by the higher value of current densities, identified along the corridors in the landscape for (a) the western block and (b) the central/eastern block of the study area.

Different CWD cutoff widths used for analysis consistently identified major pinch points at almost identical locations. However, the intensity of the corridor width changed with the increase in CWD cutoff width (S3). The CWD cutoff width of 20 km and 50 km produced very constricted corridors, whereas the CWD cutoff width of 200 km produced very wide and unrealistic corridors. We, therefore, present pinch points identified using a CWD cutoff width of 100 km. For the corridor connecting each pair of PAs, we identified several sections where the current flow was the highest, indicating higher resistance to movement for tigers, that is, pinch points or bottlenecks. Pinch points were mostly located in the periphery of the protected areas, where major highways intersected the corridor and along the Nepal–India border. Pinch points identified along the corridor connecting the Banke‐Bardia complex to Katarniaghat WLS and Shukla Phanta NP to Dudhwa NP could jeopardize future transboundary connectivity. Pinch points near PAs were particularly evident for corridors connecting Shukla Phanta NP to other PAs (Figure 5). Additionally, for the corridor connecting the Banke‐Bardia complex to Shukla Phanta NP pinch point areas were observed near the town of Chaumala and Masuriya as well as west of Lamki Bazaar (*market*) within the Siwalik landscape which may threaten the connectivity between these two important tiger recovery sites. Pinch points near major settlements and roads intersecting the corridor were also observed for other corridors identified in the landscape (Figure 5).

### 3.4 Conservation prioritization of cores and corridors

Banke‐Bardia complex was the most important PA in the landscape based on its contribution to habitat availability and maintaining tiger connectivity compared to other PAs. Despite its relatively smaller size, Shukla Phanta NP was more important for maintaining landscape connectivity based on the highest *dPC*_connector_ value (for habitat size = 28.52, for tiger population = 22.75), followed by the Banke‐Bardia complex (*dPC*_connector‐habitat_ = 27.48, *dPC*_connector‐population_ = 20.82) (S4). The contribution of protected areas within TAL‐India, except for Pilibhit TR, varied depending upon the selection of PA attribute for analysis (Table 4).

**TABLE 4:** Probability of connectivity (*dPC*) for each protected area representing its relative contribution to maintaining connectivity across the network using two different habitat node attributes.

Protected areas	Total available habitat		Estimated population	
	Attribute (~area in km^2^)	*dPC* (%)	Attribute (individuals)	*dPC* (%)
Banke‐Bardia complex	1518	28.56	150	32.99
Chitwan‐Parsa‐Valmiki complex	2481	21.21	210	26.8
Shukla Phanta NP	305	15.82	36	16.66
Pilibhit TR	1074	11.29	65	10.09
Dudhwa NP	680	8.17	20	3.91
Suhelwa WLS	452	6.01	0	0.75
Katerniaghat WLS	401	4.80	29	5.15
Nandhaur WLS	380	4.13	23	3.65

*Note*: The value of each sub‐metric for core habitats is available in the supplementary document (Table S2).

The Lagga Bhagga corridor connecting the Shukla Phanta NP to Pilibhit TR was identified as the highest‐priority linkage in terms of its contribution to maintaining landscape connectivity across both scenarios where the PAs' size and estimated tiger population were considered. The Khata corridor connecting the Banke‐Bardia complex to Katerniaghat WLS ranked second. Removing the linkages between the Chitwan‐Parsa‐Valmiki complex and the Banke‐Bardia complex and between the Bardia‐Bardia complex and Shukla NP did not affect the overall landscape connectivity (Table 5).

**TABLE 5:** Relative contribution of identified corridors to improving connectivity across the entire network based on two different habitat node attributes.

Core 1	Core 2	*dPC*_connecter_ (%) (PA size)	*dPC*_connecter_ (%) (tiger population)
Shukla Phanta NP	Pilibhit TR	41.33	44.22
Banke‐Bardia complex	Katerniaghat WLS	19.03	23.98
Shukla Phanta NP	Nandhaur WLS	16.35	17.09
Banke‐Bardia complex	Suhelwa WLS	11.27	3.61
Banke‐Bardia complex	Dudhwa NP	4.78	3.98
Shukla Phanta NP	Dudhwa NP	4.26	3.53
Chitwan‐Parsa‐Valmiki complex	Suhelwa WLS	2.98	3.61
Chitwan‐Parsa‐Valmiki complex	Banke‐Bardia complex	0	0
Banke‐Bardia complex	Shukla Phanta NP	0	0

## 4 DISCUSSION

We identified potential corridors connecting tiger‐bearing PAs in Nepal and India in the Terai Arc Landscape, one of the most important tiger conservation landscapes, by completing a landscape‐level resistance mapping analysis informed by expert knowledge. Given the dynamic landscape and ongoing threats to connectivity (Kanagaraj et al., 2013; Thapa & Tuladhar, 2021; Wikramanayake et al., 2004), our work contributes new knowledge regarding the value of corridors for tigers in the TAL and identifies a number of key changes to either refine, expand or establish new corridors in the landscape. We present an update on the current status of the landscape connectivity and, among the individual pairs of protected areas, the value of the Siwalik landscape for securing connectivity and provide the quantitative baselines for future assessment of the corridors.

Short‐distance corridors are generally expected to provide better connectivity than longer corridors (Haddad, 1999; Hilty et al., 2019) due to their relative proximity to the core habitat and the shorter time required to cross the corridor's relatively more disturbed habitat. However, the corridor connecting the Banke‐Bardia complex and the Chitwan‐Parsa‐Valmiki complex provided a relatively less costly route for tigers, despite being nearly 20 times longer than the shortest corridor in the landscape. This is mainly because the corridor passes through the Siwalik landscape's forest habitat, which offers less resistance than the habitat with large settlements and a relatively dense road network of the lowland plains for tiger movement. Forested habitats are more favorable for tigers as these provide refuge and prey resources (DNPWC & DFSC, 2018; Jhala et al., 2020). In contrast, despite being significantly shorter, the corridors originating from the Shukla Phanta NP pass through highly modified landscapes and therefore have higher cumulative resistance than other corridors, suggesting that the length of a corridor is not always a better predictor of the quality of connectivity.

The Chitwan‐Parsa‐Valmiki complex and the Banke‐Bardia complex, supporting the largest population of tigers and contiguous tiger habitat in the landscape, were most important for maintaining connectivity for tigers. In the most comprehensive global assessment of tiger landscapes, these two sites were also identified as the Level 2 priority conservation landscape for tigers, with sufficient interconnected habitats to support a breeding population of more than 50 tigers (Sanderson et al., 2010). Despite its small size, the Shukla Phanta NP was the third most important habitat patch in the landscape but also included two of the most important links for maintaining connectivity across the network. The park was previously identified as both source, and an important tiger recovery site (Harihar et al., 2018), and our analysis concurs with the finding.

Our analysis has revealed that removing corridors among the Chitwan‐Parsa‐Valimki complex, the Banke‐Bardia complex and Shukla Phanta NP did not affect the connectivity across the landscape. This is likely because the cost of directly moving among these PAs is greater for tigers than through other PAs that serve as stepping stones. For example, Suhelwa WLS, is a potential stepping stone for tigers moving from the Chitwan‐Parsa‐Valmiki complex to the Banke‐Bardia complex. Similarly, Katarniaghat WLS and Dudhwa NP are likely stepping stones for tigers moving from the Banke‐Bardia complex to Shukla Phanta NP. Improving the quality of the corridor habitat could reduce the cost of movement for tigers among the three most important core habitats for tigers in the landscape. Conservation prioritization will likely benefit the Suhelwa WLS, which currently does not support any tigers, to accommodate the tigers arriving from the landscape's two highest‐priority tiger core habitats. The prioritization of the cores and corridors presented in this paper should not be considered in isolation but in conjunction with other conservation prioritization tools.

We identified some overlap between modeled least‐cost corridors and existing corridors in the landscape but also found some potential for further optimization. For example, our analysis identified a corridor overlapping a portion of the Khata corridor with additional potential corridor habitat situated west of the Geruwa River. Previous studies also suggested that restoring 0.5 km of habitat on either side of the Khata corridor improved connectivity (Kanagaraj et al., 2013). Potential corridor habitats outside the existing boundaries were also identified for the Basanta, Laljhadi‐Mohana, Boom‐Brahmadev and Laggabagga‐Tatargunj corridors. As most of these existing corridors were reported to be non‐functional due to high levels of human disturbances (Chanchani et al., 2014; Dhakal et al., 2014; Kanagaraj et al., 2013; Thapa et al., 2017), our work makes a very important contribution to addressing the problem. Expanding existing corridor boundaries to include potential corridor habitats for better protection and habitat management efforts could improve the connectivity for tigers. Furthermore, the study highlights the method's potential in assessing and optimizing existing corridor networks that could improve connectivity.

While there is a huge potential for improving connectivity, we identified several pinch points in the landscape that, when left unaddressed, pose a threat to connectivity. Most of the pinch points were identified on the periphery of the PAs along existing transboundary corridors and where roads intersected the corridors, likely due to the high density of settlements around PAs of the TAL‐Nepal (Chanchani et al., 2014; MFSC, 2015). While tigers can disperse through human‐dominated landscapes, as they tolerate lower‐quality habitat types such as agricultural fields or open spaces when dispersing, this is only typically observed over small distances (Dutta et al., 2018). In such cases, restoring a small area of high‐quality habitat as a stepping stone along the potential movement path with higher resistance to the movement may help improve connectivity.

Pinch points introduced by road networks also pose a significant risk to connectivity for our landscape as also reported previously elsewhere (Hilty et al., 2019; Mohammadi et al., 2018). In Nepal, many settlements have been established along the roads, resulting in a very wide band of linear barriers that are highly resistant to wildlife movement. Some of the identified pinch points have long been acknowledged based on earlier research investigating tiger movements within the landscape, for example, near the town of Butwal (Smith, 1993) and along the Nepal–India border (Chanchani et al., 2014; Thapa et al., 2017). Human disturbances such as cattle grazing, fodder and firewood collection have been recorded in the Khata, Kamdi, Basanta, Laljhadi‐Mohana, Boom‐Brahmadev, and Laggabagga‐Tatargunj transboundary corridors in the past, with the potential to disrupt the movement of tigers and other wildlife through these corridors (Chanchani et al., 2014). Expanding settlements along the Terai and lower parts of the Siwalik have been identified as a major cause of forest loss and degradation in the past (Chaudhary et al., 2016; Lamichhane et al., 2018). For these corridors, pinch points were also present at agricultural and settlement areas near the corridors and along the roads passing through the corridors. Habitat restoration programs and regulating human activities within the corridor habitats are recommended to conserve landscape connectivity.

The majority of the corridors were identified through Siwalik, which is key to maintaining connectivity for the tiger in the landscape. Because of the intact forest habitat, corridors identified through the Siwalik landscapes were wider, less resistant and less likely to be severed than corridors in the lowland. Wider corridors provide more alternative routes for species movement, and even if one route is severed, species can use other alternatives, making it less susceptible to being severed. Our finding is consistent with previous studies reporting on the importance of Siwalik hills in connecting the tiger population of the TAL‐Nepal (Kanagaraj et al., 2013; Thapa & Kelly, 2017; Wikramanayake et al., 2004). However, ongoing and planned development work threatens the connectivity potential of the Siwalik. For example, a major highway spanning 1200 km through the Siwalik region is being built with little to no regard for the region's geology or potential environmental damage, with serious consequences for Terai ecology and people (NEFEJ, 2019). Nepal recently adopted the federal governance structure, thereby equipping local and provincial governments with the financial capacity and authority to meet their respective local communities' development needs and aspirations. However, the absence of coherent development plans across local governments, coordination among local agencies, and expertise in integrating environmental concerns into development projects (Bhattarai et al., 2018; Thakali et al., 2018) may contribute to further degradation of the Siwalik landscape and its ability to support landscape connectivity.

As with any other ecological model, our analysis is subject to errors resulting from untested assumptions of the connectivity model, uncertainties associated with the habitat selection by species during movement and dispersal, and the accuracy of spatial data layers in representing actual landscape conditions. Assessment of tiger corridors in the landscape through other available methods such as resistant kernel density, circuitscape, factorial least‐cost path and based on data sourced from genetic, telemetry, and species distribution studies would be useful to improve corridor identification further. Nonetheless, we anticipate that the corridors identified in our analysis will be useful for policymakers, conservation practitioners, and researchers in prioritizing connectivity conservation initiatives in the Siwalik landscape and informing future research and planning to secure landscape connectivity in the study area.

## 5 CONCLUSION

Securing connectivity for the tiger has been recognized as a major conservation action needed to ensure the species' long‐term survival (DNPWC, 2016; GTI, 2011; Jhala et al., 2021). Our connectivity model based on expert knowledge and spatial environmental data serves as a necessary starting point for identifying potential connectivity between tiger‐bearing PAs across a large heterogeneous landscape in the TAL‐Nepal. Similar approaches and knowledge syntheses have been used recently in India to identify agreement among stakeholders to prioritize connectivity conservation efforts (Schoen et al., 2022). Despite the rapid conversion of forest habitats in the lowland in recent decades (Ram et al., 2021; Reddy et al., 2018), the TAL's forest in the Siwalik region is relatively intact and provides a unique opportunity to maintain connectivity among PAs. There are opportunities for optimization of the existing network of corridors in the landscape by extending protection and habitat management interventions to considerable habitats outside established PAs and corridors suitable for improving landscape connectivity. Also, several pinch points along the identified corridors in the landscape require immediate conservation attention to maintain potential connectivity.

The corridors identified in our study have implications for local, national, and global tiger conservation efforts and government obligations toward the Convention of Biological Diversity, sustainable development goals, and several other conservation and climate change frameworks to which the government is a party. Conservation of these potential corridors identified between PAs of Nepal through increased protection status, habitat restoration, limiting impacts of infrastructure development, and habitat encroachment and resource exploitation will help the government of Nepal to achieve the national targets for tiger and protected area conservation (DNPWC, 2016, 2022) and those envisioned in the Strategy and Action Plan for Terai Arc Landscape (2015–2025), Nepal (MFSC, 2015) and other similar landscape‐level conservation initiatives. Similarly, the pinch points identified along transboundary corridors can help prioritize habitat restoration activities to protect existing connectivity from further deterioration and the long‐term persistence of tigers in the globally significant tiger conservation landscape in addition to the identified priority core and corridors. Future studies using empirical data and other connectivity modeling methods and including other connectivity‐dependent species are recommended to guide corridor planning and implementation in the landscape.

## AUTHOR CONTRIBUTIONS

**Tek Bhatt**: Conceptualization‐Lead, Formal analysis‐Lead, Methodology‐Lead, Writing – original draft‐Lead, Writing – review & editing‐Equal. **Guy Castley**: Conceptualization‐Equal, Formal analysis‐Equal, Supervision‐Equal, Writing – review & editing‐Equal. **Rebecca Sims‐Castley**: Data curation‐Equal, Formal analysis‐Equal, Methodology‐Supporting, Software‐Supporting, Writing – review & editing‐Equal. **Hem Baral**: Conceptualization‐Equal, Formal analysis‐Equal, Supervision‐Equal, Writing – review & editing‐Equal. **Ali Chauvenet**: Conceptualization‐Equal, Formal analysis‐Equal, Supervision‐Equal, Writing – review & editing‐Equal.

## FUNDING INFORMATION

Not Applicable.

## CONFLICT OF INTEREST STATEMENT

The authors declare that they have no competing interests.

## Supporting information


**Data S1:** Supporting Information

## Data Availability

The data that supports the findings of this study are available in the article and supplementary material of this article. These data were derived from the resources available in the public domain the link to which is provided in the manuscript.
