# Supplementary material for: Connecting tiger (Panthera tigris) populations in Nepal: Identification of corridors among tiger‐bearing protected areas
Source: Ecol Evol. 2023 May 30;13(5):e10140. doi: 10.1002/ece3.10140 (PMC10227491; doi:10.1002/ece3.10140)
Supplement: Supplementary file 1 — Data S1: Supporting Information [file ECE3-13-e10140-s001.pdf]

Connecting Tigers in Nepal: identification of corridors between tiger-bearing protected areas.

S1 Land cover classification and accuracy assessment

In the absence of a recent national landcover map for Nepal, we created a landcover layer by performing multi-spectral classification using publicly-available Landsat 8 Operational Land Imager (OLI) satellite imagery at 30m resolution. We downloaded cloud-free (<10% cloud cover), atmospherically corrected imagery for Oct- Nov 2019 from the USGS portal (https://earthexplorer.usgs.gov), as this period follows the monsoon season when cloud cover is at its lowest, resulting in optimal spectral coverage of vegetation. Our study area was captured in six separate scenes (Path/Row: 141/41, 142/41, 143/40, 143/41, 144/40, 145/40), which were processed, classified, and analyzed in ERDAS Imagine 14.0 and ArcGIS 10.6 (ESRI, Redlands, CA, USA) to produce thematic maps identifying the following broad a priori landcover classes: forest, water bodies, agriculture, barren areas, and urban areas.

After an initial exploration of the data using ISODATA and principal component analysis (PCA) to identify six bands (bands 1-6) for analysis, a supervised classification using a maximum likelihood algorithm was applied to each scene. After each classification run, an accuracy assessment of the classification was performed by comparing landcover classes to 300 randomly sampled ground reference points for each scene.

Accuracy assessment is an important step of the image classification process to ensure that the image classification represents the actual land cover. We assessed our classification accuracy for each scene by generating 300 random points called ground reference points. We identified the actual land cover class for each ground reference point using google earth imagery for 2019-2020. We then created an error matrix for our classification by comparing actual landcover classes to the landcover classes predicted in the classified image for each ground reference point.

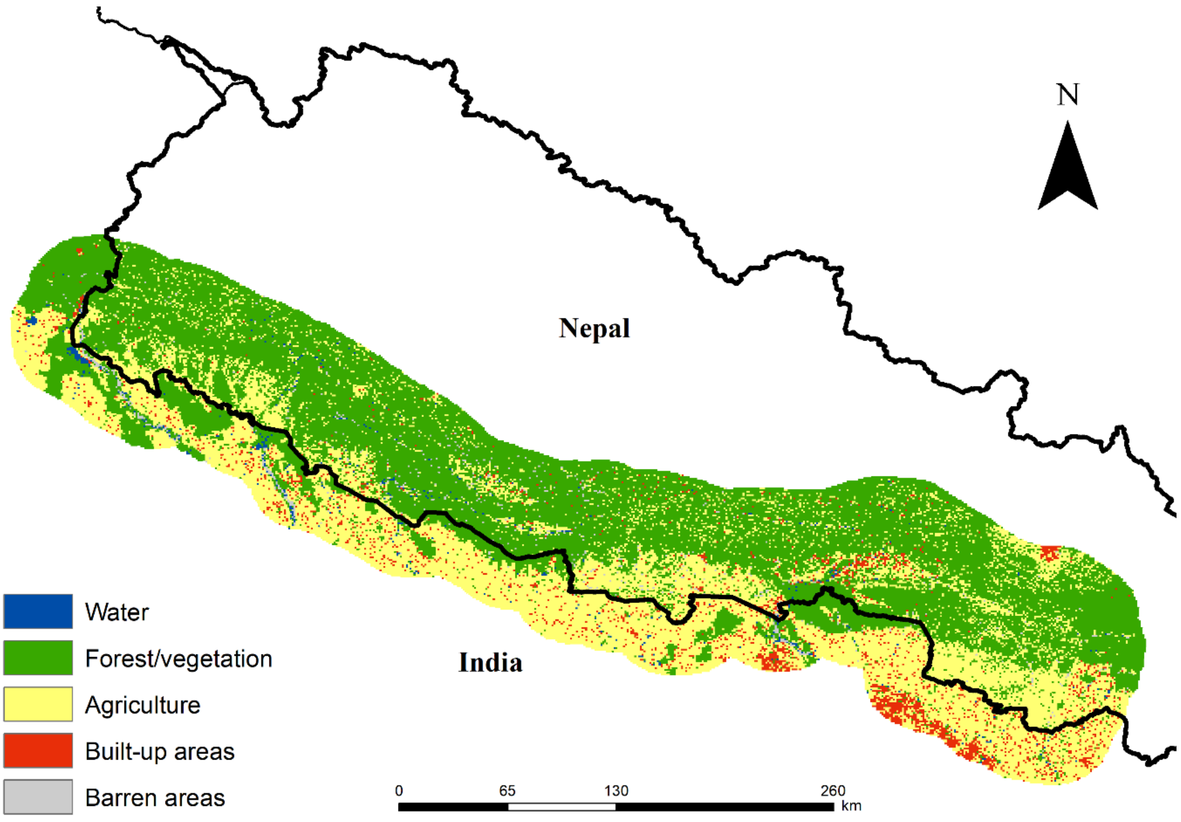

Fig. S1.1 Landcover map developed for the study area with broad habitat classes used for creating resistance surface

We calculated the user's, the producer's and the overall accuracy, and the kappa coefficient for each scene. Individual classified scenes were then mosaicked to get the landcover classification of the study area. We repeated the classification for several trials, improving the training data sample each time until the overall accuracy for each scene was above 80% and the kappa coefficient value >0.6. We also assessed the final image's accuracy and calculated the user's accuracy, the producer's accuracy, the overall accuracy, and the kappa coefficient. User's accuracy refers to the error of commission when an incorrect pixel is added to the landcover class; producer's accuracy refers to the error of omissions when a correct pixel is not included in the landcover class.

The forest was the major habitat type (55%), followed by agriculture (39%), urban (4%), barren areas (>1%), and water (<1%) in our study area (Fig. 3). The majority of the forest (~65%) of

the study area was found in hilly regions above 250 m elevations, whereas more than 85% of the agricultural land and 90% of the human settlement area of the study area were found below 250 m elevation. The lowland region also had the highest density of human populations and road networks. The overall accuracy for the landcover classification of the study area was 82%, with a kappa coefficient of 0.6.

Table S1.1 Error Matrix for overall land cover classification

Landcover classes	Water	Forest	Agriculture	Urban	Barren	**Total**	User's Accuracy	producer's accuracy
Water	15	2	0	1	2	**20**	0.750	0.536
Forest	5	724	102	4	3	**838**	0.864	0.919
Agriculture	7	58	172	10	5	**252**	0.683	0.621
Urban	1	0	3	18	5	**27**	0.667	0.545
Barren	0	4	0	0	22	**26**	0.846	0.595
**Total**	**28**	**788**	**277**	**33**	**37**	**1163**		

Overall accuracy: 0.82

Kappa coefficient: 0.6

S2 Comparison of resistance surfaces derived from environmental variables, different spatial scales, and weighting scenarios

The ability of connectivity models to correctly predict corridors for target species is determined by how well the resistance surface, the fundamental unit of connectivity modeling, reflects "true" landscape conditions. Some environmental variables may influence tiger movement more than other environmental variables. Weighting allows one to reflect these relative differences in influence by each environmental variable in the model, i.e. weighting a variable higher than another allows it to have more influence on the model outcomes. The selection of spatial scale (grain or pixel size) and relative weighting of environmental variables are also important factors that can affect the ability of resistance surfaces to predict corridors (Zeller et al., 2012). Fig. 2 provides an overview of the workflow process carried out.

We compared the resistance surfaces resulting from different combinations of spatial scale and layer weighting scenarios to identify the most appropriate resistance surface for our analysis. We obtained the resistance scores for each environmental variable on a scale of 0-100 through an expert opinion survey. We used Gnarly Landscape Utilities (McRae et al., 2013) to create the resistance surfaces and the Linkage Mapper tool (McRae & Kavanagh, 2011) in ArcGIS to map the least cost corridors.

a) Identification of spatial scale for corridor analysis

Objective: Assess the influence of the spatial scale of input data on corridor modeling and identify the most appropriate scale for the study landscape.

Method: To identify the most appropriate spatial scale for our analysis, we resampled the 30m raster grid cell or pixel size of each environmental variable to 100 m (SS1), 250 m (SS2), 500 m (SS3), 1 km (SS4), 2 km (SS5), and 4 km (SS6) and, with each environmental input layer weighted equally. Resistance surfaces were calculated at each of the new spatial scales respectively. We added 1 to account for Euclidean distance (the minimum cost to travel a given

pixel). The resultant resistance surfaces were analyzed using the Linkage Pathway tool within the Linkage Mapper toolbox (McRae and Kavanagh, 2011), run via ArcGIS, to map cost-weighted distance (CWD) corridors between PAs, where CWD indicates the cumulative cost of movement to any given point in the landscape from the nearest core habitat using individual resistance surfaces. A CWD threshold of 200,000 m was used to delineate the corridor area (Dutta et al., 2018).

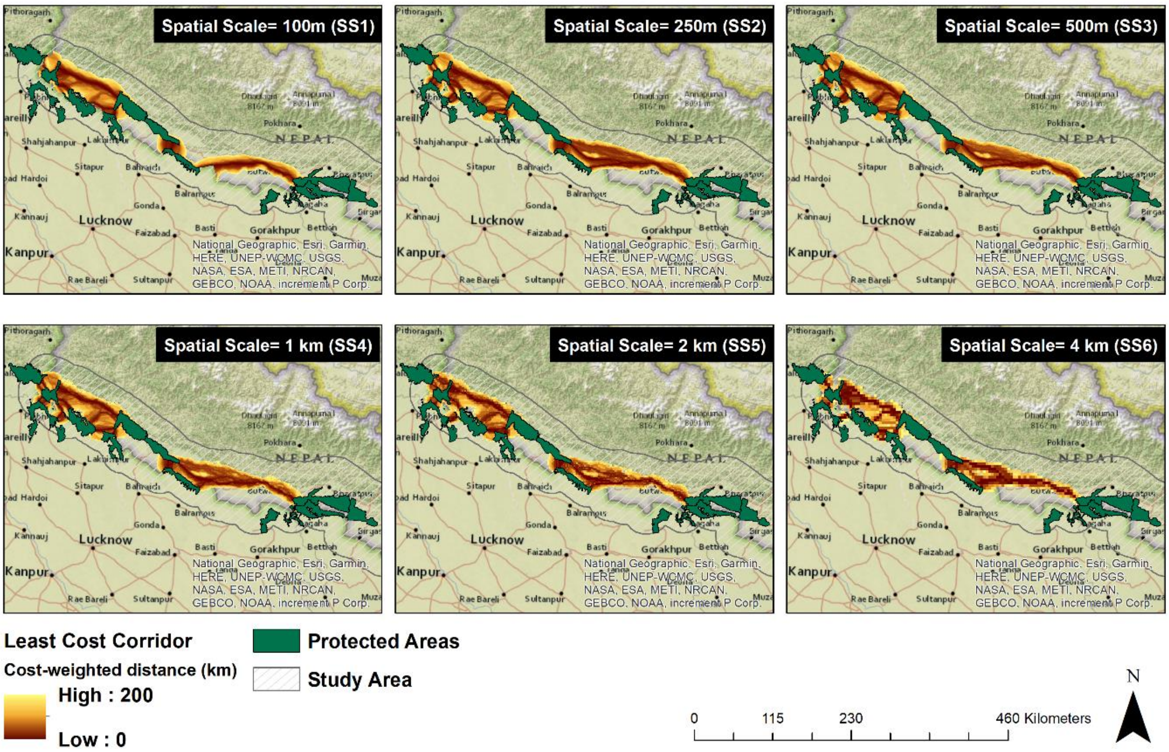

Fig. S2.1 Corridor models using resistance surfaces generated from input data at different spatial scales. The spatial scale of analysis for each map is given at the top of the respective map.

We first resampled all corridors to 100m resolution to compare the corridor outputs from different resistance surfaces. We then identified the overlap between individual corridors to get a consensus corridor across six models by selecting those pixels classified as a corridor in five or more models. The consensus corridor represents the area of the landscape considered most important for connectivity by most corridors derived from individual spatial scale scenarios.

The spatial scale, which produces the most similar corridor to the consensus corridor, was then identified as the optimal spatial scale for analysis. We compared the individual corridor model with the consensus corridor for spatial similarity by calculating Jaccard Similarity Index (JSI) (Arponen et al., 2012; Tomaselli et al., 2013). We selected the spatial scale of the corridor model (SS4, 1 km) most similar to the consensus corridor, with the highest similarity score of 0.94. Jaccard Similarity Index was calculated as:

$$\text{Jaccard Similarity Index (JSI)} = \frac{A \cap B}{A \cup B}$$

Where A and B are two entities to be compared.

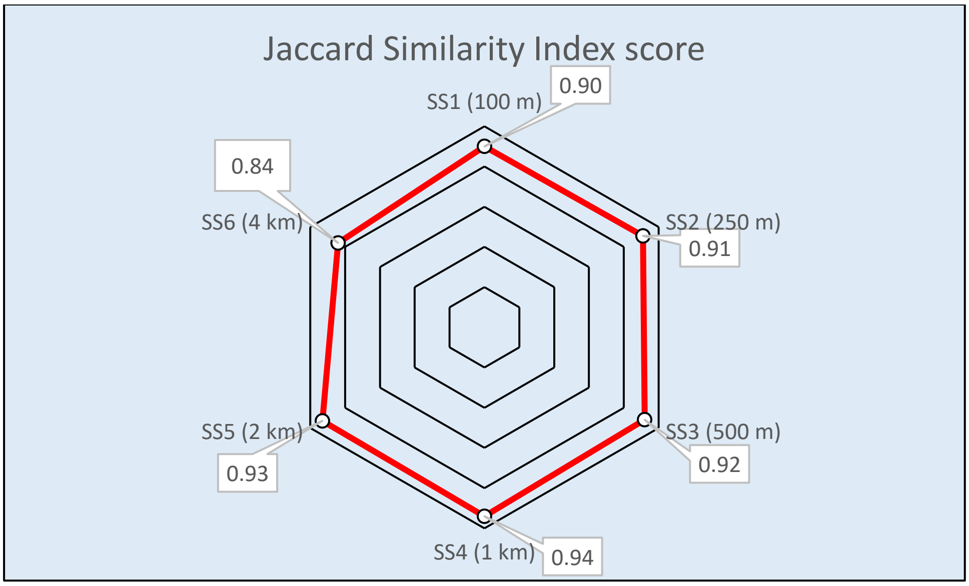

Fig. S2.2 Jaccard Similarity Index score of different resistance surfaces (SS1-SS6) with consensus raster

b) Identification of optimal layer weighting scenarios for corridor analysis

Objective: Assess the influence of different layer weightage scenarios on corridor modeling and identify the most appropriate scenarios for corridor analysis.

Method: Once the optimal spatial analysis scale had been established, it was then possible to determine the ideal layer weighting. This was achieved by applying different sets of weightings to each environmental variable to calculate the resistance surfaces and then comparing the resultant least-cost corridor outputs. To this end, we evaluated the effects of each set of weighting scenarios by comparing four Run Options (RO) where various weights were applied to each variable. The definition of ROs was based on studies conducted by Rathore et al. (2012) and Dutta et al. (2018), both of which identified the greater influence of landcover and anthropogenic variables on tiger movement. For our study, we compared the following layer weighting scenarios: i) all variables have equal importance (RO1), ii) landcover has twice the importance of other variables (RO2), iii) both landcover and human population density have twice the importance of other variables (RO3), and iv) landcover, human population density, and road network are equally important, but the slope is not included (RO4).

Run Option 1 (RO1) = Landcover + Human population density + Road network + Slope

Run Option 2 (RO2) = 2 Landcover + Human population density + Road network + Slope*

Run Option 3 (RO3) = 2(Landcover + Human population density) + Road network + Slope*

Run Option 4 (RO4) = Landcover + Human population density + Road network + 0 Slope*

A resistance surface was calculated using each weighted environmental variable for each RO. As with the spatial scale evaluation, resultant resistance outputs were used to generate CWD corridor maps (using Linkage Mapper). Applying the same 200,000m CWD cutoff to delineate the corridor area, a consensus corridor was identified by overlapping the resulting corridors for

each RO. We calculated a similarity score between each RO and consensus corridor using the Jaccard Similarity Index. The corridor resulting from the RO scenario with the highest similarity score was selected as the final corridor for further analysis and interpretation. This enabled us to present the least-cost corridors and pinch points identified at the most optimal spatial scale and the best combination of the layer-weighting scenario for our study landscape.

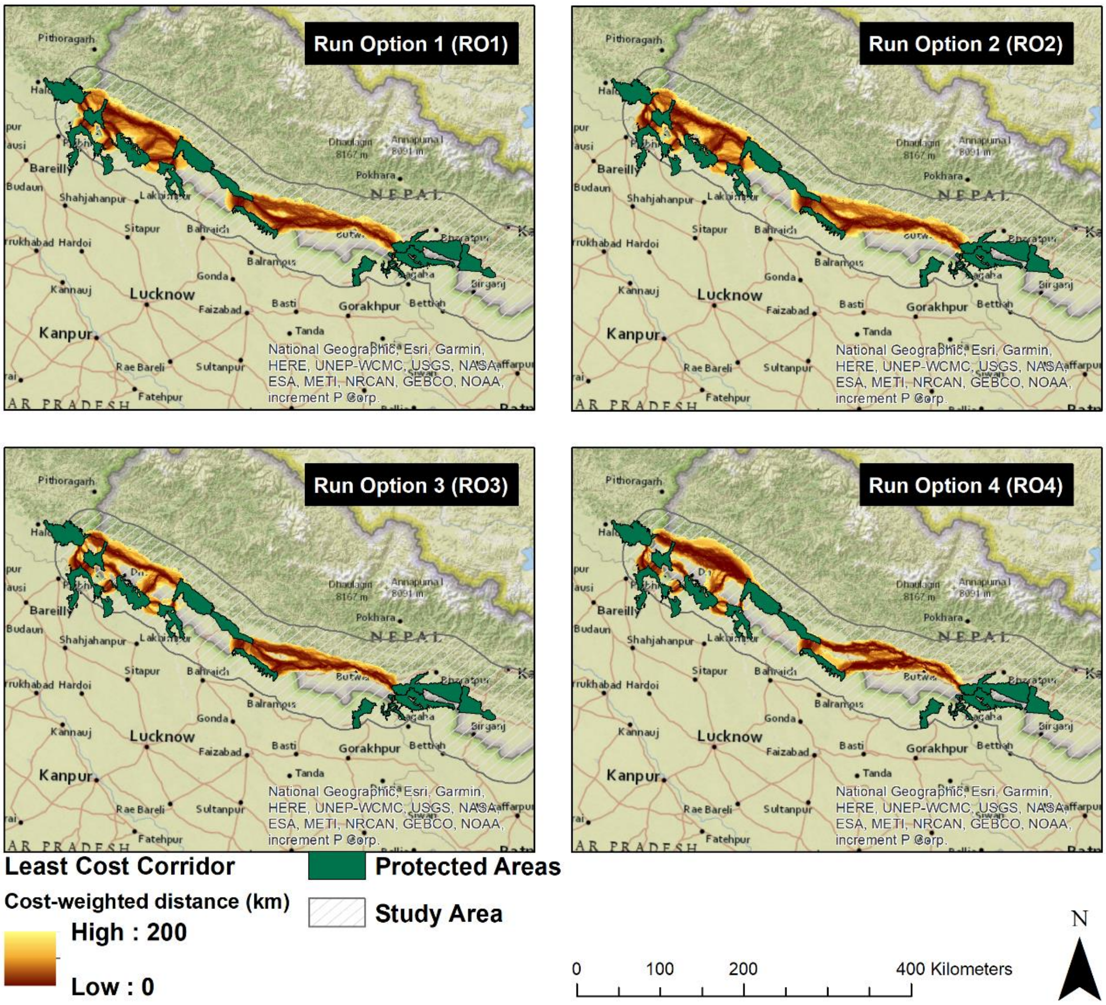

Fig. S2.3 Corridor models from resistance surfaces using different layer weighting scenarios. Reference to the layer weighting scheme is given at the top of each map

We identified a consensus corridor across four models by selecting the area classified as the corridor in at least three models. We then compared the individual corridor model with the

consensus corridor by calculating Jaccard Similarity Index. We selected the layer weighting scheme (RO3) most similar to the consensus corridor.

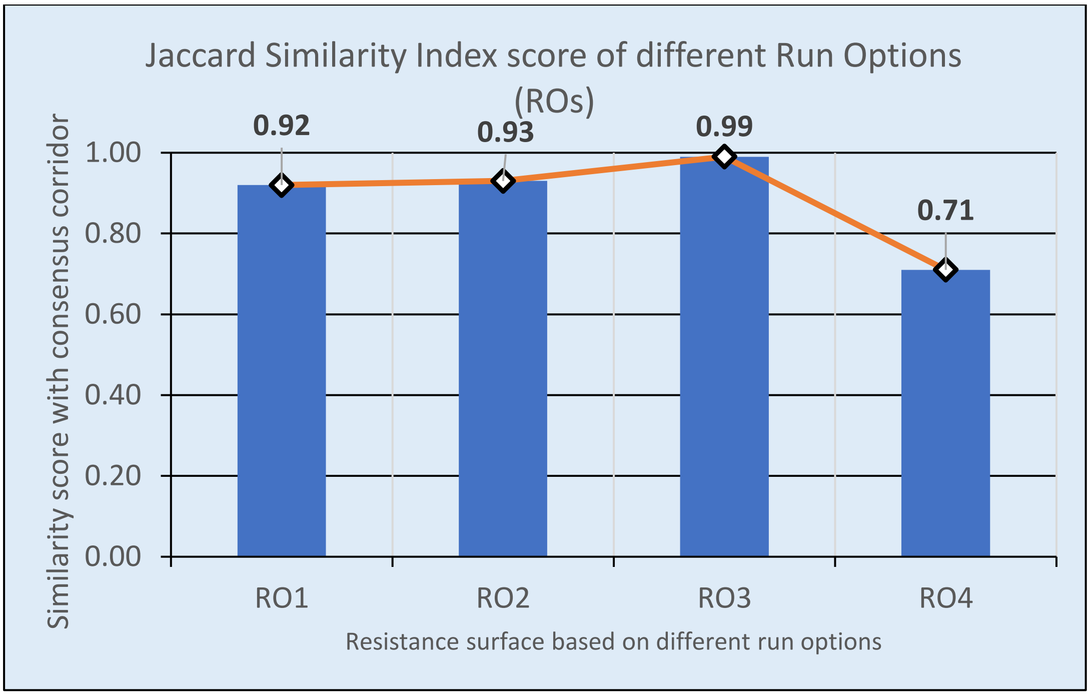

Fig. S2.4 Jaccard Similarity Index score of different resistance surfaces with consensus corridor raster

We found that the selection of different spatial scales and weighting scenarios of the input data altered the position of the least-cost path and corridor identified in the landscape. This was particularly evident at very fine (100 m) or coarse spatial scales (4 km). However, there was little difference among corridor outputs from resistance surfaces generated at 250 m, 500 m, 1 km, and 2 km spatial scales, suggesting that a wide range of spatial scales of input data layers could be used for identifying corridors for tigers in the landscape. A similar pattern of considerable agreement was observed among corridors identified in Central India using resistance surfaces derived from various sources, spatial and temporal scales, and parameterizations across multiple studies, though they used a circuit theory approach different from this study's methodological approach. (Schoen et al., 2022). Our finding is consistent with the previous suggestion that the coarse spatial scale better represents the scale at which highly

mobile terrestrial mammal species, such as large carnivores, interact with the environment (Galpern et al., 2012). The thematic resolution (the categories or levels of environmental variables), another factor reported to dominate the effect of both spatial grain and extent in generating resistance surface (Cushman & Landguth, 2010), was not used in the sensitivity analysis. Assessing the effect of different thematic resolutions for environmental variables in future studies may further help develop a more accurate resistance surface for the tigers in the landscape.

S3 Comparision of cutoff width for pinch point analysis

Following Dutta et al., (2016), we compared different corridor cutoff widths of 20 km, 50 km, 100 km and 200 km CWD. Major connectivity pinch points were identified at identical locations across different cutoff widths. In such cases, it has been suggested that a more generous cutoff width be selected, especially if the study is conducted at a coarse spatial scale that could yield a wider but more feasible linkage zone. It also addresses some of the underlying uncertainties associated with GIS input data, resistance model, and parameters used for corridor analysis (Dutta et al., 2016; WHCWG, 2010). In their assessment, Dutta et al., (2016) used a threshold of at least 50% forest cover within the corridor to identify the appropriate cutoff width for presenting the result of the pinch point analysis. We also applied a similar approach but used a threshold of 80% because our landscape has comparatively more forest cover (>55%) than the Central Indian Landscape (>33%) (Dutta et al., 2018). The proportion of forest habitat across the corridor across cutoff widths of 20 km, 50 km, 100 km and 200 km was 93%, 87%, 81% and 76%, respectively. Corridor containing a certain proportion of habitats other than the forest, such as agricultural or barren lands, is more likely to occur in the real world and still be functional, as tigers can tolerate slightly disturbed habitat, especially during dispersal. Therefore, we selected 100 km cutoff width, with >80% of forest cover within the corridor, to present the results of the pinch point analysis.

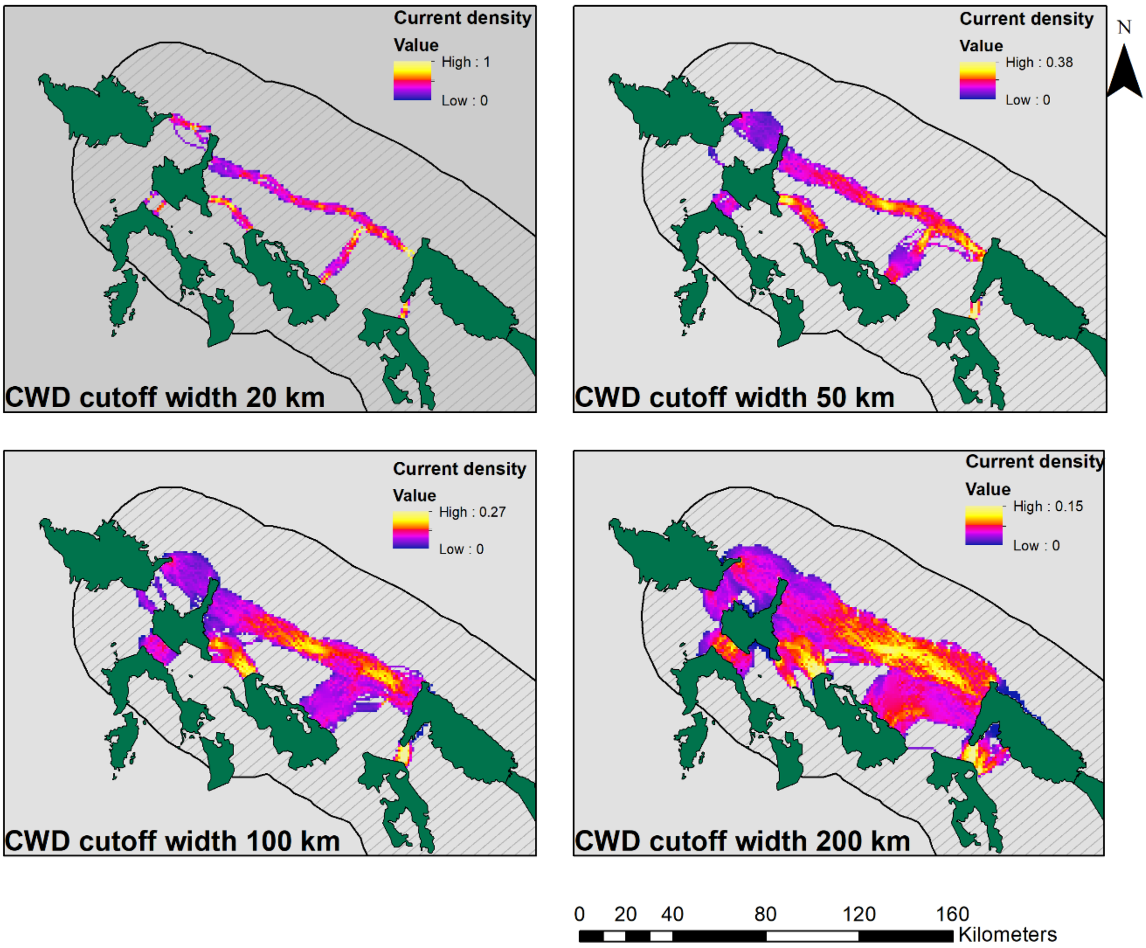

Fig. S3.1(a) Effects of different corridor cutoff widths on pinch point analysis results (showing pinch points along the corridor connecting PAs of the western part of the landscape)

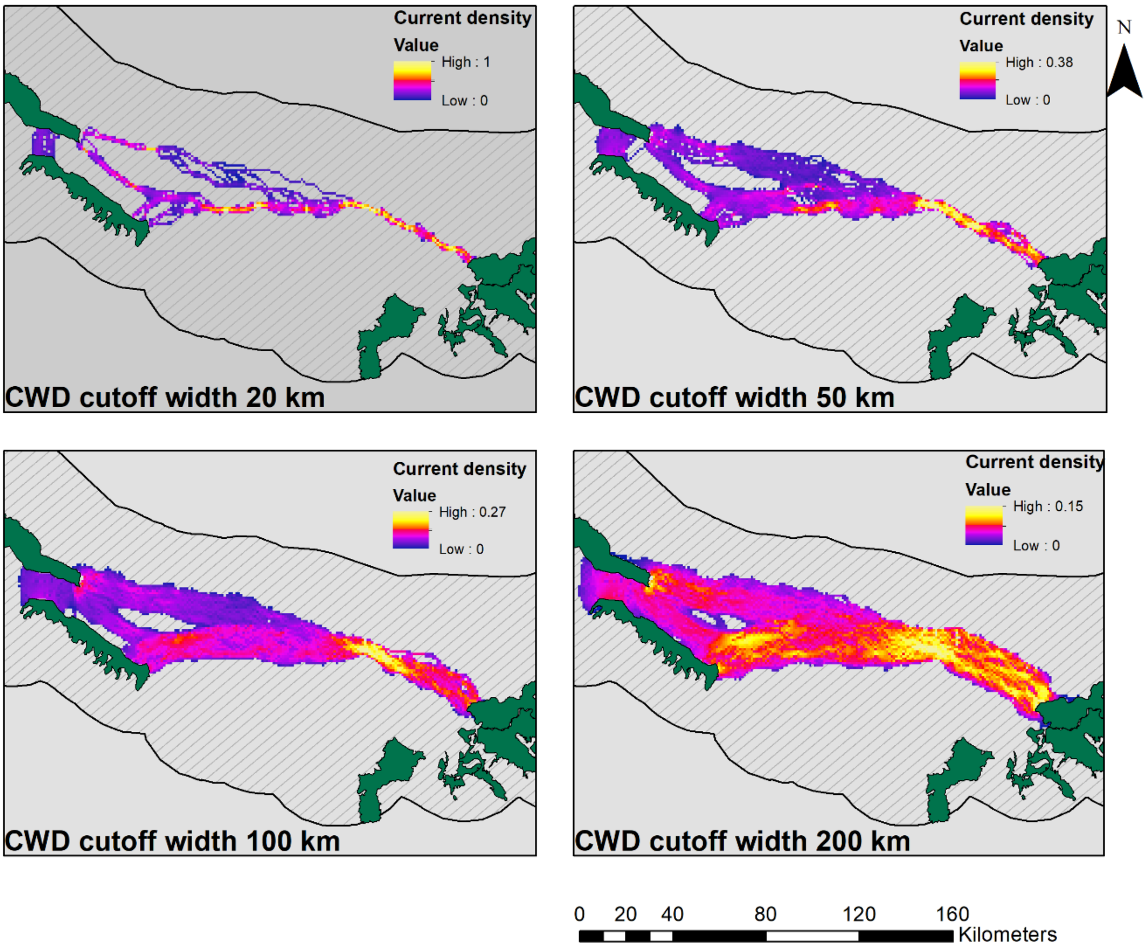

Fig. S3.2 (b) Effects of different corridor cutoff widths on pinch point analysis results (showing pinch points along the corridor connecting PAs of the western part of the landscape)

S4 Conservation prioritization of cores and corridors

We assessed the importance of each PA (nodes) and the corridors (links) using a graph-theoretic approach to assist with their conservation prioritization for maintaining the landscape connectivity. For this, we used Conefor v2.6 (Saura & Torné, 2009), which takes PAs (i.e. any habitat patch with defined attributes as nodes) and corridors (links connecting a pair of nodes) as input to produce indices useful to rank the contribution of individual nodes and connections to habitat availability and connectivity (Saura & Torné, 2009; Saura & Rubio, 2010). We used each PA core's estimated tiger population size for the node attribute and the cost-weighted distance among each pair of PAs obtained from the least-cost corridor analysis for links. We calculated the probability of connectivity *(dPC)* to assess the importance of the nodes and links in the landscape. We considered five different Euclidean dispersal distance thresholds (30 km, 50 km, 100 km, 150 km and 175 km) for tigers in the landscape, assuming a dispersal probability of 50% for calculating the probability of connectivity at each threshold. The average CWD per unit EuCD (~29) among the PA pairs was used to get distance thresholds in CWD (870 km, 1450 km, 2900 km, 4350 km and 5075 km). The minimum and maximum dispersal thresholds were selected based on the previously reported maximum dispersal distance for adult tigers, i.e., 30 km (Smith, 1993) and the maximum Euclidean distance among any pairs of PAs in the landscape (= ~173 km between Chitwan NP and Banke NP).

Table S4.2: Probability of connectivity score for each protected area representing its relative contribution to maintaining connectivity across the network at varying dispersal distance thresholds. The estimated tiger population was used as a habitat node attribute.

PA cores	***dPC (30km)***	***dPC (50km)***	***dPC (100km)***	***dPC (150km)***	***dPC (175km)***
Banke-Bardia complex	*45.26*	*54.36*	*64.72*	*68.71*	*69.87*
Chitwan-Parsa-Valmiki complex	*44.81*	*46.87*	*52.75*	*55.8*	*56.76*
Shukla Phanta NP	*21.06*	*26.85*	*32.47*	*34.64*	*35.29*
Pilibhit TR	*16.06*	*18.32*	*20.35*	*21.13*	*21.36*
Katerniaghat WLS	*10.14*	*10.75*	*10.98*	*10.94*	*10.91*
Dudhwa NP	*7.95*	*8.79*	*8.67*	*8.39*	*8.28*
Nandhaur WLS	*5.28*	*6.34*	*7.28*	*7.63*	*7.74*
Suhelwa WLS	*1.27*	*2.15*	*2.14*	*1.75*	*1.58*

The relative importance of the individual PAs for maintaining connectivity across all PAs was highest for the Banke Bardia complex across all dispersal thresholds. The importance of the PAs increased with an increase in the dispersal distance thresholds. For protected areas relatively close to others eg. Katarniaghat WLS, the importance of the habitat cores for maintaining connectivity remained stable across increasing thresholds of dispersal distance. This implies that the maximum importance of a habitat patch can be gained if a suitable habitat patch is restored within the dispersal threshold distance.

Table 4.3: The probability of connectivity and its fraction values for each core habitat based on two different attributes used for analysis with a dispersal threshold of 175 km Euclidean distance.

PA Core	Habitat size				Population size			
	dPC	dPC_{intra}	dPC_{flux}	$dPC_{connector}$	*dPC*	dPC_{intra}	dPC_{flux}	$dPC_{connector}$
Banke-Bardia complex	66.19	5.72	32.99	27.48	69.87	10.46	38.59	20.82
Chitwan-Parsa-Valmiki	49.16	15.28	33.88	0.00	56.76	20.50	36.26	0.00
Shukla Phanta NP	36.66	0.23	7.91	28.52	35.29	0.60	11.93	22.76
Pilibhit TR	26.17	2.86	23.31	0.00	21.36	1.96	19.40	0.00
Dudhwa NP	18.95	1.15	16.87	0.92	8.28	0.19	7.12	0.97
Suhelwa WLS	13.92	0.51	12.09	1.32	1.58	0.00	0.00	1.58
Katerniaghat WLS	11.13	0.40	10.73	0.00	10.91	0.39	10.52	0.00
Nandhaur WLS	9.58	0.36	9.22	0.00	7.74	0.25	7.50	0.00

References

Arponen, A., Lehtomäki, J., Leppänen, J., Tomppo, E., & Moilanen, A. (2012). Effects of connectivity and spatial resolution of analyses on conservation prioritization across large extents. *Conservation Biology, 26*(2), 294-304. doi:https://doi.org/10.1111/j.1523-1739.2011.01814.x

Cushman, S. A., & Landguth, E. L. (2010). Scale dependent inference in landscape genetics. *Landscape Ecology, 25*(6), 967-979. doi:https://doi.org/10.1007/s10980-010-9467-0

Dutta, T., Sharma, S., McRae, B. H., Roy, P. S., & DeFries, R. (2016). Connecting the dots: mapping habitat connectivity for tigers in central India. *Regional Environmental Change, 16*(0), 53-67. doi:https://doi.org/10.1007/s10113-015-0877-z

Dutta, T., Sharma, S., & DeFries, R. (2018). Targeting restoration sites to improve connectivity in a tiger conservation landscape in India. *PeerJ, 6*, e5587. doi:https://doi.org/10.7717/peerj.5587

Galpern, P., Manseau, M., & Wilson, P. (2012). Grains of connectivity: analysis at multiple spatial scales in landscape genetics. *Molecular Ecology, 21*(16), 3996-4009. doi:https://doi.org/10.1111/j.1365-294X.2012.05677.x

McRae, B. H., & Kavanagh, D. M. (2011). Linkage mapper connectivity analysis software. Retrieved from https://circuitscape.org/linkagemapper/

McRae, B. H., A.J. Shirk, & Platt., J. T. (2013). Gnarly Landscape Utilities: Resistance and Habitat Calculator User Guide. The Nature Conservancy, Fort Collins, CO. Retrieved from https://circuitscape.org/gnarly-landscape-utilities/

Rathore, C. S., Dubey, Y., Shrivastava, A., Pathak, P., & Patil, V. (2012). Opportunities of habitat connectivity for tiger (*Panthera tigris*) between Kanha and Pench National Parks in Madhya Pradesh, India. *PLoS ONE, 7*(7), e39996. doi:https://doi.org/10.1371/journal.pone.0039996

Saura, S., & Torné, J. (2009). Conefor Sensinode 2.2: A software package for quantifying the importance of habitat patches for landscape connectivity. *Environmental Modelling & Software, 24*(1), 135-139. doi:https://doi.org/10.1016/j.envsoft.2008.05.005

Saura, S., & Rubio, L. (2010). A common currency for the different ways in which patches and links can contribute to habitat availability and connectivity in the landscape. *Ecography, 33*(3), 523-537. doi:https://doi.org/10.1111/j.1600-0587.2009.05760.x

Schoen, J. M., Neelakantan, A., Cushman, S. A., Dutta, T., Habib, B., Jhala, Y. V., et al. (2022). Synthesizing habitat connectivity analyses of a globally important human-dominated tiger-conservation landscape. *Conservation Biology, 36*(4), e13909. doi:https://doi.org/10.1111/cobi.13909

Smith, J. L. D. (1993). The role of dispersal in structuring the Chitwan tiger population. *Behaviour, 124*(3-4), 165-195. doi:https://doi.org/10.1163/156853993X00560

Tomaselli, V., Dimopoulos, P., Marangi, C., Kallimanis, A. S., Adamo, M., Tarantino, C., et al. (2013). Translating land cover/land use classifications to habitat taxonomies for landscape monitoring: a Mediterranean assessment. *Landscape Ecology, 28*(5), 905-930. doi:https://doi.org/10.1007/s10980-013-9863-3

WHCWG. (2010). *Washington Connected Landscapes Project: Statewide Analysis*.W. W. H. C. W. Group, USA. Retrieved from https://waconnected.org/wp-content/themes/whcwg/docs/statewide-connectivity/Chapter%202%20-%20Methods%20WHCWG%20Statewide%20Analysis.pdf

Zeller, K. A., McGarigal, K., & Whiteley, A. R. (2012). Estimating landscape resistance to movement: a review. *Landscape Ecology, 27*(6), 777-797. doi:https://doi.org/10.1007/s10980-012-9737-0
